# Process-Driven Modulation of Structure and Cytocompatibility in Electrospun Silk Fibroin Membranes through Controlled Degumming

**DOI:** 10.1021/acsomega.6c04899

**Published:** 2026-08-22

**Authors:** K.K. Gómez-Lizárraga, E. Robles-Hurtado, H.A. Méndez-Escobar, C. Velasquillo, Stephen Muhl Saunders, J. Cruz-Cárdenas, R. Vera-Graziano

**Affiliations:** † Programa Investigadoras e Investigadores por México. Secretaría de Ciencia, Humanidades, Tecnología e Innovación / Instituto de Investigaciones en Materiales, Universidad Nacional Autónoma de México, Circuito Exterior S/N. Circuito de la Investigación Científica, Ciudad Universitaria, Ciudad de México 04510, México; ‡ Instituto de Investigaciones en Materiales, 130323 Universidad Nacional Autónoma de México, Circuito Exterior S/N. Circuito de la Investigación Científica, Ciudad Universitaria, Ciudad de México 04510, México; § Unidad de Ingeniería de Tejidos, Terapia Celular y Medicina Regenerativa, Instituto Nacional de Rehabilitación Luis Guillermo Ibarra Ibarra, Ciudad de México 14389, México; ∥ Facultad de Ciencias, Universidad Nacional Autónoma de México, Ciudad de México 04510, México

## Abstract

The degumming process technique critically determines the structural and biological performance of silk fibroin-based biomaterials. In this study, silk fibroin obtained from *Bombyx mori* cocoons was subjected to a controlled experimental design that combined sodium carbonate (Na_2_CO_3_), sodium lauryl ether sulfate (SLES), and reaction time to modulate the efficiency of sericin removal. Degumming effectiveness was quantified by mass loss and chemical analysis (FTIR-ATR, solid-state ^13^C NMR), while structural modifications were evaluated by X-ray diffraction. Alkaline-based degumming treatments (C4–C9) achieved sericin removal efficiencies above 90%, promoted β-sheet exposure, and resulted in higher crystallinity compared to surfactant-only treatments. Regenerated silk fibroin was processed by solution electrospinning under controlled conditions. The electrospun porous membranes exhibited predominantly random-coil conformations, with crystallinity values ranging from 24–38%. Cytocompatibility assessment using human dermal fibroblasts demonstrated a concentration-dependent response. The morphology of the electrospun fibers, including surface roughness and wettability (SEM, microindentation, and contact angle measurements), led to adequate adhesion of normal fibroblasts. Additionally, the mechanical properties, including hardness, Young’s modulus, and elongation, provide suitable elastic properties for fibroblast proliferation. Membranes derived from alkaline-treated fibroin showed cell viability above 80%, while surfactant-only treatments exhibited reduced metabolic activity at full extract concentration. No intrinsic antibacterial activity was observed against *Staphylococcus aureus* or *Pseudomonas aeruginosa*. These findings demonstrate that the degumming strategy and electrospinning conditions are key upstream parameters regulating structure–property-cytocompatibility relationships in electrospun silk fibroin membranes. Optimal alkaline degumming and electrospinning conditions enhance biological safety and reproducibility for tissue engineering applications.

## Graphical abstract

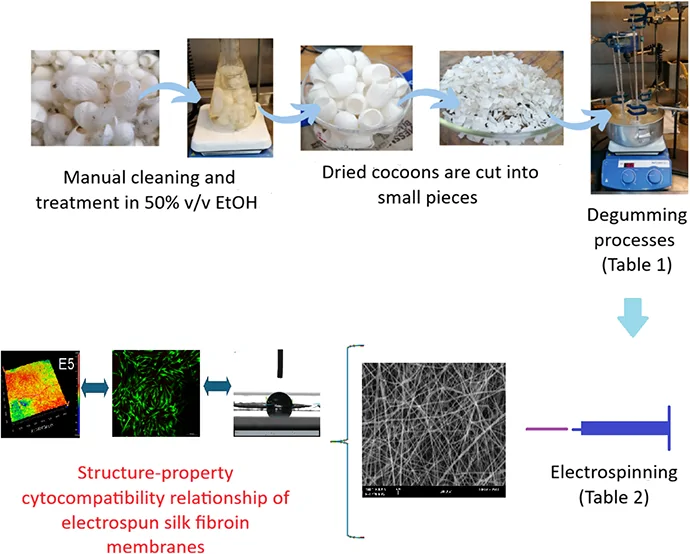

## 1 Introduction

Silk fibroin derived from *Bombyx mori* cocoons has been widely investigated as a biomaterial for drug delivery, wound healing, and tissue engineering due to its mechanical robustness, biocompatibility, and tunable biodegradability.^1,2^ Native silk consists primarily of two proteins: fibroin (≈72–83%) and sericin (≈17–28%), where sericin forms a hydrophilic coating surrounding fibroin filaments.

Silk fibroin forms two types of crystals, silk I and silk II. The first one has a helicoidal or random-coil structure, which facilitates water solubility. In contrast, silk II is water-insoluble due to its antiparallel β-sheet structure, intermolecular bonds between chains (disulfide bonds), and stabilization by hydrogen bonds.

Silk sericin, on the other hand, is composed of polar side-chain amino acids, including serine (Ser), asparagine (Asp), and threonine (Thr), with a decreasing concentration from the outer to the inner layer. SS is considered a glue-like protein because it holds two fibroin fibers together as a coating.

For biomedical applications, sericin must be removed via degumming to expose fibroin and ensure structural stability and biocompatibility. Various degumming approaches have been reported, including surfactant treatments,^3^ alkaline^4^ and acidic solutions,^5^ and enzymatic methods^6^ and boiling water.^7,8^ Among them, pH, degumming agent concentration, high temperatures, and processing time must be controlled; otherwise, these factors can affect secondary structures, disrupt hydrogen bonds, and even degrade molecules.

Despite extensive use of silk fibroin in electrospun scaffolds, limited attention has been paid to how degumming parameters influence downstream structural organization and biological responses.

Thus, once sericin has been removed, fibroin can be dissolved and processed (regenerated silk fibroin, RSF) into films, sponges, or nanofibers. RSF can be processed into scaffolds by depositing fibers in the micro- and nanometer ranges, which can be randomly oriented or aligned, presenting a large surface area. These scaffolds are known as electrospun nanofibers, produced by electrospinning.^9–17^

Electrospinning enables the fabrication of nanofibrous membranes with high surface area and an extracellular matrix–mimicking architecture.^18–22^ The structural characteristics of electrospun fibers are strongly dependent on solution chemistry and molecular organization, which in turn are influenced by prior processing steps. Therefore, degumming conditions may critically affect not only fibroin purity but also the development of secondary structure, crystallinity, and subsequent cytocompatibility.

In this work, a controlled experimental design combining alkaline and surfactant treatments was implemented to modulate sericin removal. The central hypothesis is that the degumming strategy and electrospinning conditions serve as structure-regulating parameters that govern crystallinity and cytocompatibility in electrospun silk fibroin membranes. Structural characterization was correlated with in vitro biological response to establish processing–structure–property relationships relevant to tissue engineering applications.

## 2 Materials and Methods

### 2.1 Materials

Silkworm cocoons were donated by the Center for Molecular Biology and Biotechnology of the Technological University of Pereira, Colombia. Trifluoroacetic acid (TFA), sodium lauryl ether sulfate (SLES), sodium carbonate (Na_2_CO_3_), and ethyl alcohol (Sigma-Aldrich, St. Louis, MO, USA). Calcein-green AM (Molecular Probes, Eugene, OR, USA) and propidium iodide (PI) reagents.

For the biological test, a Hanks’ balanced (HBSS; Gibco) salt solution (with composition mM (125 NaCl, 0.9 KCl, 3.6 NaHCO_3_, 0.3 Na_2_HPO_4_, 0.4 KH_2_PO_4_, 0.5 MgCl_2_, 0.4 MgSO_4_, 10 glucose, 2.9 sucrose, 9.9 HEPES, and 2.2 CaCl_2_, pH 7.2) was used.

### 2.2 Degumming Process

The silkworm cocoons (*Bombyx mori* *)* were manually cleaned and treated with a 50% v/v ethanol solution for 30 min under continuous stirring.

A degumming process was then performed to expose the silk fibroin. The solutions used for this purpose were an aqueous alkaline solution of Na_2_CO_3_, a surfactant solution of SLES, and a mixture of both Na_2_CO_3_ and SLES reagents in varying proportions to remove the sericin coating.

The degumming process was performed at 80 °C at different concentrations and reaction times, as shown in Table 1.

**Table 1:** Description of the Conditions of the Different Degumming Processes (the C1 Sample Was Treated Only with Boiling Water)

Process	Na_2_CO_3_ (%w/V)	SLES (%w/V)	Time (min)
C1	0	0	30
C2	0	0.25	60
C3	0	0.5	90
C4	0.25	0	60
C5	0.25	0.25	90
C6	0.25	0.5	30
C7	0.5	0	90
C8	0.5	0.25	30
C9	0.5	0.5	60

For each degumming process, approximately 1 g of cocoon was used. After degumming, all samples were thoroughly washed with deionized water to remove residual reagent, then dried and weighed to determine the amount of dissolved sericin.

The percent mass loss after each degumming process was calculated according to eq 1:^23^

1 $$Mass\;loss\;(\%)=\left(\frac{m_{before\;degumming}-m_{after\;degumming}}{m_{before\;degumming}}\right)\times 100$$

The degumming process efficiency was determined as the percentage of mass loss, with the C4 process considered 100% degumming efficacy (eq 2).

2 $$Degum\min g\;efficiency:\frac{{mass\;loss}_{process}}{{mass\;loss}_{C4}}\times 100$$

### 2.3 Electrospinning Process

Silk fibroin solutions (or RSF) were prepared in TFA at 3% and 5% w/v concentrations and stirred for 2 h at room temperature. The electrospinning parameters for the fabrication of the scaffolds used corresponded to a Taguchi design of experiment (statistical analysis in Figure S1, Supporting Information) with a needle diameter of 22 and 33 gauge (G), a flow rate of 0.5 and 1 mL/h, a collector distance of 15 and 20 cm, and a voltage of 15 and 20 kV (see Table 2).

**Table 2:** Electrospinning Parameters of Silk Fibroin Solution

Scaffold	Degumming sample	Concentration (% w/V)	Voltage (kV)	Collector distance (cm)	Flow rate (mL/h)	Needle diameter (G)
E1	C2	3	15	15	0.5	22
E2	C2	5	20	20	1	33
E3	C3	3	15	15	0.5	22
E4	C3	5	20	20	1	33
E5	C4	3	15	15	0.5	22
E6	C4	5	20	20	1	33
E7	C5	3	15	15	0.5	22
E8	C5	5	20	20	1	33
E9	C6	3	15	15	0.5	22
E10	C6	5	20	20	1	33
E11	C7	3	15	15	0.5	22
E12	C7	5	20	20	1	33
E13	C8	3	15	15	0.5	22
E14	C8	5	20	20	1	33
E15	C9	3	15	15	0.5	22
E16	C9	5	20	20	1	33

### 2.4 Morphology and Surface Characterization

The morphology of both the exposed fibroin fibers and the electrospun membranes was analyzed by scanning electron microscopy (SEM) following degumming. The samples were sputter-coated with a thin layer of conductive material and observed using a JEOL field-emission microscope (JSM-7600F). ImageJ software was used to measure the fiber diameters of the scaffolds.

A 3D noncontact optical profilometer (Zygo NewView, Zygo Corp., CT, USA) was used to measure the surface roughness of the electrospun membranes. For each sample, four points were measured, and the average roughness parameter (S_a_) was determined. A field of view of 747 × 747 μm was measured using a 10× objective and a 2× multiplier.

This technique was also used to measure the penetration length in the microindentation test on the samples, based on the diagonal formed by the indenter geometry mark.

The wettability of the electrospun surface was measured using a contact angle instrument (KRÜSS ADVANCE, Germany). A drop-sessile method was employed with a water drop volume of 4 μL, and the results were analyzed over time. The average contact angle was calculated from three measurements of each sample.

A dimensional retention test was performed under wet conditions; 6 mm-diameter circles from each electrospun membrane were used in quadruplicate. The samples were placed in culture wells, and distilled water was added and maintained at 37 °C to simulate physiological conditions. At 24, 48, and 72 h, the liquid was removed. The percentage change in area was calculated before and after each defined time point, as shown in the eq 3:

3 $$\%CA=\frac{A_{0}-A}{A_{0}}\times 100$$

Where A_0_ is the area of the circle before wet treatment, and A is the area after immersion in distilled water. ImageJ software was used to measure the samples.

### 2.5 Chemical Composition Characterization

#### 2.5.1 Fourier-Transformed Infrared Spectroscopy with Attenuated Total Reflectance (FTIR-ATR)

The chemical composition of the silkworm cocoon, degumming samples (C1 to C9), and electrospun membranes (E1 to E16) was determined by FTIR-ATR (Nicolet 6700, Thermo Fisher, Waltham, MA, USA) coupled with a diamond accessory for ATR, in a wavelength range between 400 and 4000 cm^–1^, with a resolution of 4 and 8 cm^–1^ and 32 scans. OriginPro software was applied to process the data.

#### 2.5.2 ^13^C Nuclear Magnetic Resonance

The chemical structure of the silk fibroin fiber after the degumming process was analyzed by Nuclear Magnetic Resonance (^13^C NMR). The experiment was performed in a solid-state configuration using a Bruker Avance III HD 400 MHz NMR spectrometer at 5000 Hz with a 4 mm HR-MAS probe. The spectra were analyzed using the MestRenova v14.0 software.

#### 2.5.3 X-ray Diffraction (XRD)

The crystalline structure of fibroin membranes was analyzed by XRD with a Bruker D8 Advance diffractometer using a Cu K-alpha 1 (λ = 1.5406 Å) radiation source operating at 30 kV and 30 mA. X-ray diffraction analysis was performed over a range of 10° to 50° at a scanning rate of 1°/min.

The crystalline percent (%) of the scaffolds (eq 4) was estimated by integrating the area corresponding to the crystalline proportion (peak, A_C_) between the total area (peak and the inclusion of the amorphous part, A_A_) as follows:^24^

4 $$crystalline\;\%=\frac{A_{C}}{A_{C}+A_{A}}\times 100$$

Figure S2 shows the processing of raw data from the diffractograms of each sample in Origin (version 8).

### 2.6 Mechanical Properties

A microindentation test was performed using a NANOVEA mechanical tester to determine the elastic-plastic properties of the electrospun membranes, which preserved fibroblast cellular morphology. A Vickers diamond indentation tip was pressed against the samples at a load of 5 N, and a loading/unloading rate of 0.25 N/min (with a hold of 20 s), and the depth of penetration was recorded. Three indentations per sample were performed to study reproducibility.

Through the set of elements between the indenter and the specimen, the reduced modulus (E_r_) is determined using eq 5:

5 $$\frac{1}{E_{r}}=\frac{1-\nu_{s}^{2}}{E_{s}}+\frac{1-\nu_{i}^{2}}{E_{i}}$$

Where ν_s_ (0.33,^25,26^ ) and E_s_ are the Poisson’s ratio and elastic modulus of the specimen, and ν_i_ and E_i_ are those corresponding to the indenter. For soft materials, the indenter term could be neglected, and eq 5 is reduced to eq 6, also known as the effective modulus:^27^

6 $$E_{r}=\frac{E_{s}}{1-\upsilon_{s}^{2}}$$

From eq 6, the Young’s modulus of the specimen is obtained. Considering the value of Er determined from the equation described by the Oliver and Pharr method^28^ using the following equation (eq 7)­

7 $$E_{r}=\frac{\sqrt{\pi }}{2}\frac{S}{\sqrt{A}}$$

Here, S is the contact stiffness between the indenter and the specimen obtained from the slope of the linear fit of the unloading curve, and A is the contact area based on the indenter geometry.

Meanwhile, the hardness is calculated from eq 8:^29^

8 $$H=\frac{F}{S}$$

Where F is the maximum load.

### 2.7 Electrospun Membranes Preparation for Cell Culture

#### 2.7.1 Scaffold Sterilization

Briefly, electrospun membranes (5 × 5 mm^2^) were immersed in Dulbecco’s modified Eagle’s medium (DMEM) (Sigma-Aldrich Co.) supplemented with 1% (v/v) Antibiotic–Antimycotic solution (containing 100 U/mL penicillin, 100 μg/mL streptomycin, and 0.25 μg/mL amphotericin B) Gibco, Thermo Fisher Scientific, followed by ultraviolet (UV) irradiation for 10 min per side (20 min total exposure). After sterilization, the membranes were rinsed twice with sterile PBS to remove residual antimicrobial agents. Throughout the manuscript, the term “sterilized membranes” refers specifically to membranes treated with this protocol.

#### 2.7.2 Cell Viability Assay

Primary dermal human adult fibroblasts obtained from the American Type Culture Collection (ATCC PCS-201-012) were used for all assays. Cells were cultured with Fibroblast Basal Medium (ATCC, USA, PCS-201-030) supplemented with 10% FBS and with a fibroblast growth kit (ATCC PCS-201-041), and maintained in an incubator at 37 °C, 80% humidity, and 5% CO_2_. The cell line was maintained in supplemented Fibroblast Basal Medium (ATCC, USA, PCS-201-030). All cells were cultured in 75 cm^2^ tissue culture flasks (Corning, USA) at 37 °C in a humidified 5% CO_2_ atmosphere. Subconfluent cells were subcultured every day until they reached 80% confluence. Cells were seeded in triplicate over 96-well culture dishes with and without a membrane.

Drops of 50 μL containing 4 × 10^3^ fibroblasts were evenly seeded on every electrospun membrane, to allow cell adhesion. Constructs (scaffolds and cells) were incubated in fibroblast-supplemented medium for 1 h. Following incubation, constructs were completely immersed in the final volume of 200 μL in supplemented medium for 24 h and maintained in an incubator at 37 °C, 80% humidity, and 5% CO_2_.

After 24 h, the culture medium was removed, and cells were incubated for 30 min in Hank’s balanced salt solution (HBSS; Gibco) with 2 μM Calcein AM and 2 μM Ethidium homodimer (EthD-1). Calcein fluorescent signal was observed with a fluorescein band-pass filter and EthD-1 with a Texas Red filter. Images were captured and analyzed using a confocal microscope (LMS 880, Carl Zeiss).

#### 2.7.3 Cytotoxicity Assay

**I**n vitro cytotoxicity testing was performed using the MTT assay (3-(4,5-dimethyl thi- 24 azolyl-2) 2,5-diphenyltetrazolium bromide (Thermo Fisher Scientific, Oregon). The cytocompatibility of fibroblasts on the sterilized membranes was evaluated using supernatant dilutions (lixiviates) in accordance with ISO 10993-5.

Briefly, sterilized membranes were previously incubated with agitation at 37 °C in supplemented culture medium (DMEM-F12/10% FBS/1% antibiotic–antimycotic) for 24 h. After this, liquid extracts were diluted in supplemented media to obtain the working concentrations tested: 100, 50, 25, 12.5, and 0% v/v.

Besides, fibroblasts were seeded in 96-well cell culture plates at a density of 1 × 10^4^ cells per well and cultured for 24 h. Fibroblasts were exposed to 20% v/v ethanol solution (SDS, Sigma-Aldrich) at the same time point as the dead cell cytotoxic control.

At each incubation time, supernatants were removed from the wells, replaced with 100 μL of MTT solution (0.5 mg/mL), and incubated at 37 °C for 3 h. Formazan crystals were dissolved in a 1:1 isopropanol/dimethyl sulfoxide (DMSO) solution and transferred to a new 96-well plate. Absorbance was measured using a Spectro-photometer Multiskan GO (Thermo Scientific, Pittsburgh, PA) at 570 nm, and cell viability was calculated with the following formula (eq 9):

9 $$\%\;Cell\;viability=\frac{I_{s}}{I_{C}}\times 100$$

Where, I_s_ is the absorbance of the cells exposed to the extract, and I_c_ is the absorbance of the cells not exposed to the extract.

### 2.8 Antibacterial Activity Test

The antibacterial activity of electrospun fibroin/sericin membranes was tested against *Staphylococcus aureus* *(* *S. aureus* *)* ATCC 25923 and *Pseudomonas aeruginosa* *(* *P. aeruginosa* *)* ATCC 27853, which are Gram-positive and Gram-negative bacteria, respectively.

Both strains were propagated on 5% sheep blood agar under aerobic conditions for 18 h. One colony of each strain was inoculated in 5 mL of tryptic soy agar medium (BD Bioxon) and allowed to incubate for 18 h at 37 °C with stirring at 220 rpm.

Sterilized membranes at each concentration were inoculated with the corresponding bacteria and incubated in triplicate at 37 °C. A positive control (without scaffolds) and a negative control (without bacteria) were used to determine the antibacterial capacity of the electrospun membranes. After 18 h, the optical density (OD600 nm) was measured using a spectrophotometer at 600 nm, based on the turbidity of the suspensions.

### 2.9 Statistical Analysis

Statistical analyses were performed using GraphPad Prism 9.5.0 (Dotmatics, Boston, USA) and Origin (Northampton, MA 01060, USA) software. Data were expressed as the mean ± SD unless stated otherwise. Statistical significance was determined using ANOVA. The results were considered to be statistically significant when *p* < 0.05.

## 3 Results and Discussion

### 3.1 Degumming Process

The quantification of sericin coating removal was determined as the percentage of mass loss of the degummed silkworm cocoon, representing the amount of sericin dissolved in the solutions. In the literature, it has been reported that the sericin content in silk is approximately 25–30% by weight.^30,31^ Therefore, as shown in Figure 1a, samples C4 to C9 are free from sericin. Meanwhile, sample C1, treated only with boiling water, shows a weight loss of around 6% due to sericin. Degumming processes of C2 and C3 were treated with 0.25% and 0.5% w/v of SLES, respectively. Both exhibit behavior similar to C1, with a slight increase in sericin loss during degumming in C3.

**Figure 1:**
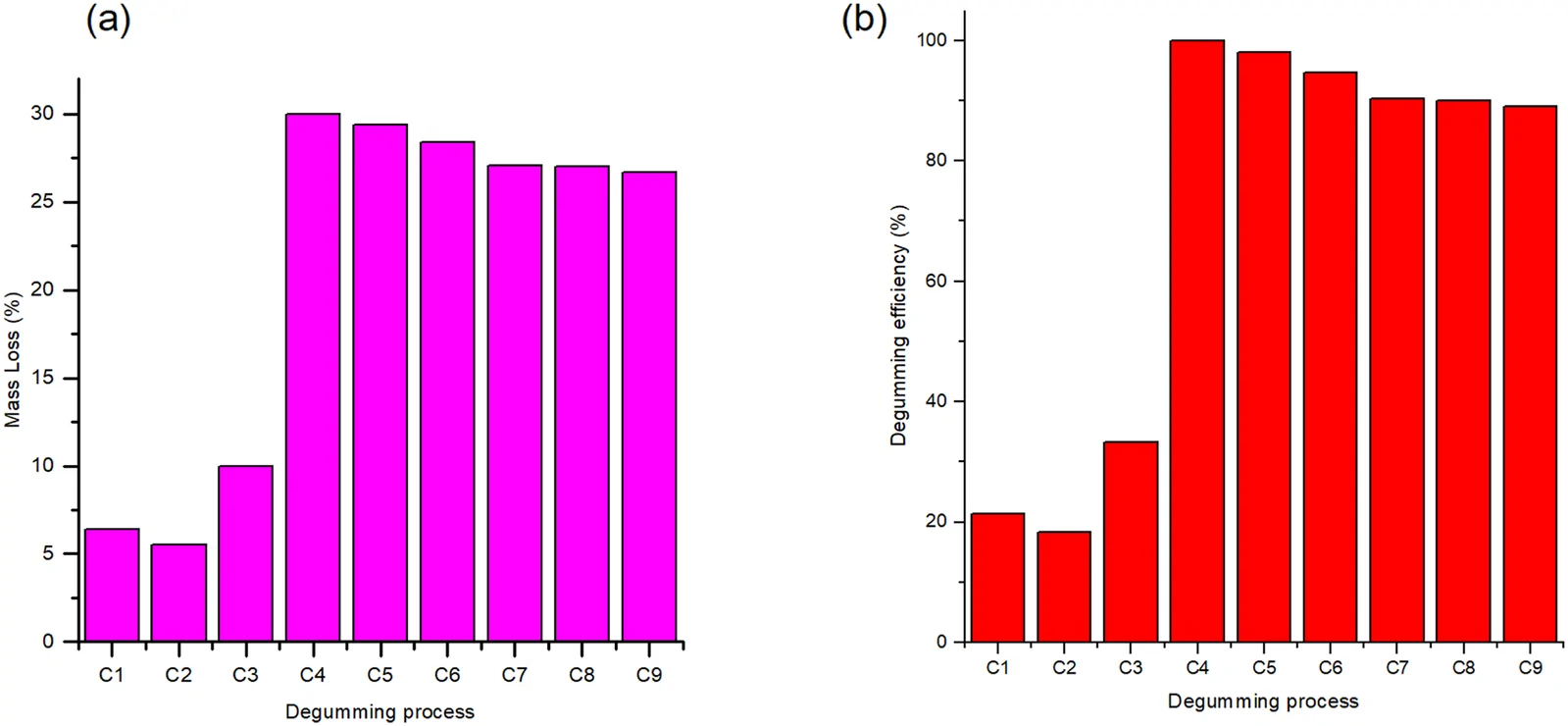
Percentage of mass loss associated with (a) the removal of sericin and (b) the degumming efficiency of the nine processes.

C4 and C5 showed a high sericin weight loss, almost 30%. The contribution of SLES in the degumming solution depletes the solubility of sericin. In combination with carbonate, it has a minimal effect on the degumming process.

The same behavior is observed in the degumming efficiency (Figure 1b), where C4 to C9 show the highest percentages, exceeding 90%. For the first degumming process, the efficiency ranges from 18% to 35%. Based on this result, an alkaline solution showed better performance in removing sericin from silk fibers.

The morphology of silkworm cocoons was analyzed before (Figure 2A) and after the degumming process (Figure 2B), as shown in Figure 2. After the degumming process (Figure 2B), a clear separation between the fibroin fibers is evident, as the sericin content has been removed. This micrograph contrasts with that before degumming (Figure 2A), where the sericin covering the fibroin fibers was removed.

**Figure 2:**
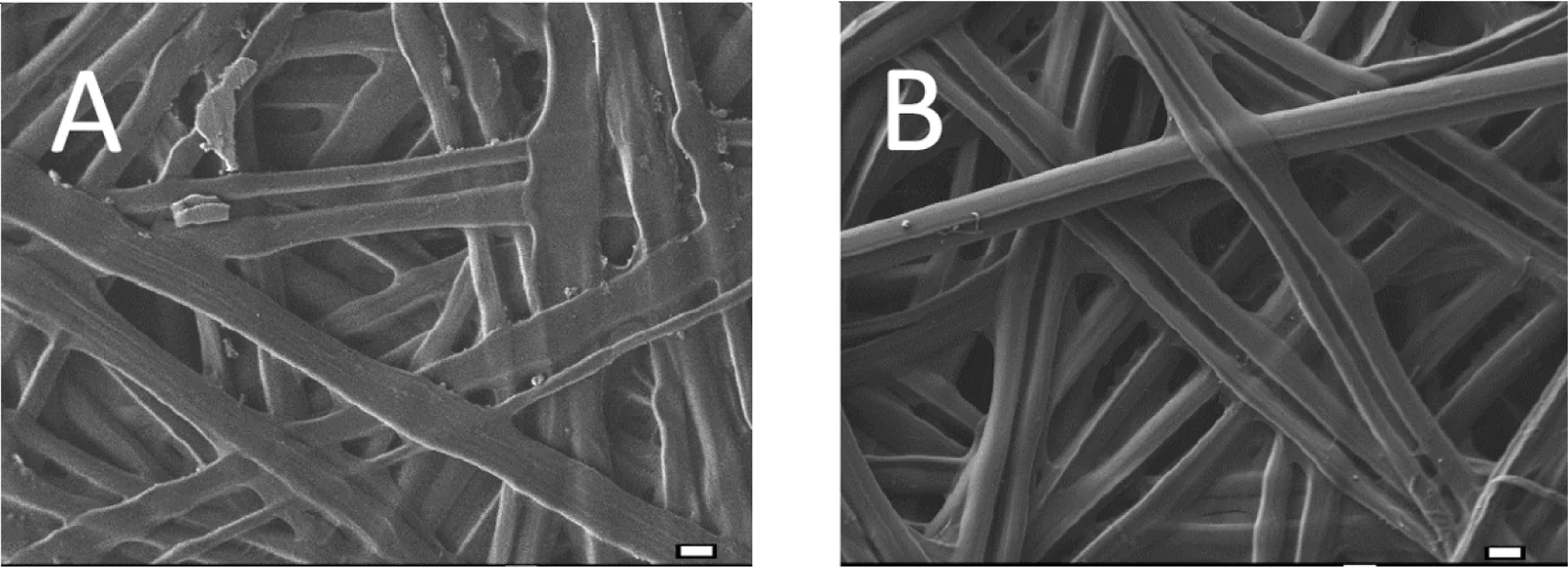
Morphology of silkworm cocoon before degumming (A) and after degumming (B). Bar indicated 10 μm.

The micrographs in Figure S3 show that the degummed silk fibroin, RSF (C1–C9), has a smooth surface, also suggesting sericin removal, in comparison to the native silkworm cocoon fibers, which exhibit a rough surface.

### 3.2 Chemical Composition by FTIR-ATR

The chemical composition of *B. mori* silk cocoon fibers and degummed silk cocoon.

The molecular conformation associated with the secondary structures of the protein in the *B. mori* silkworm is influenced by the interaction of Amide bonds through carbonyl and/or amine functional groups of the protein and between solvent molecules mediated by hydrogen bonds.

From the FTIR-ATR spectra in Figure 3, the main absorption bands of silk are the Amide A, B, I, II, and III vibrational modes, characteristic of the protein backbone. Amide A and B appear around 3275 cm^–1^ and 3072 cm^–1^, respectively. Both are due to N–H stretching vibration modes. According to Wöltje, M. et al.^32^ the Amide I, II, and III can be described in terms of secondary conformations: random-coil and β-sheet structures.

**Figure 3:**
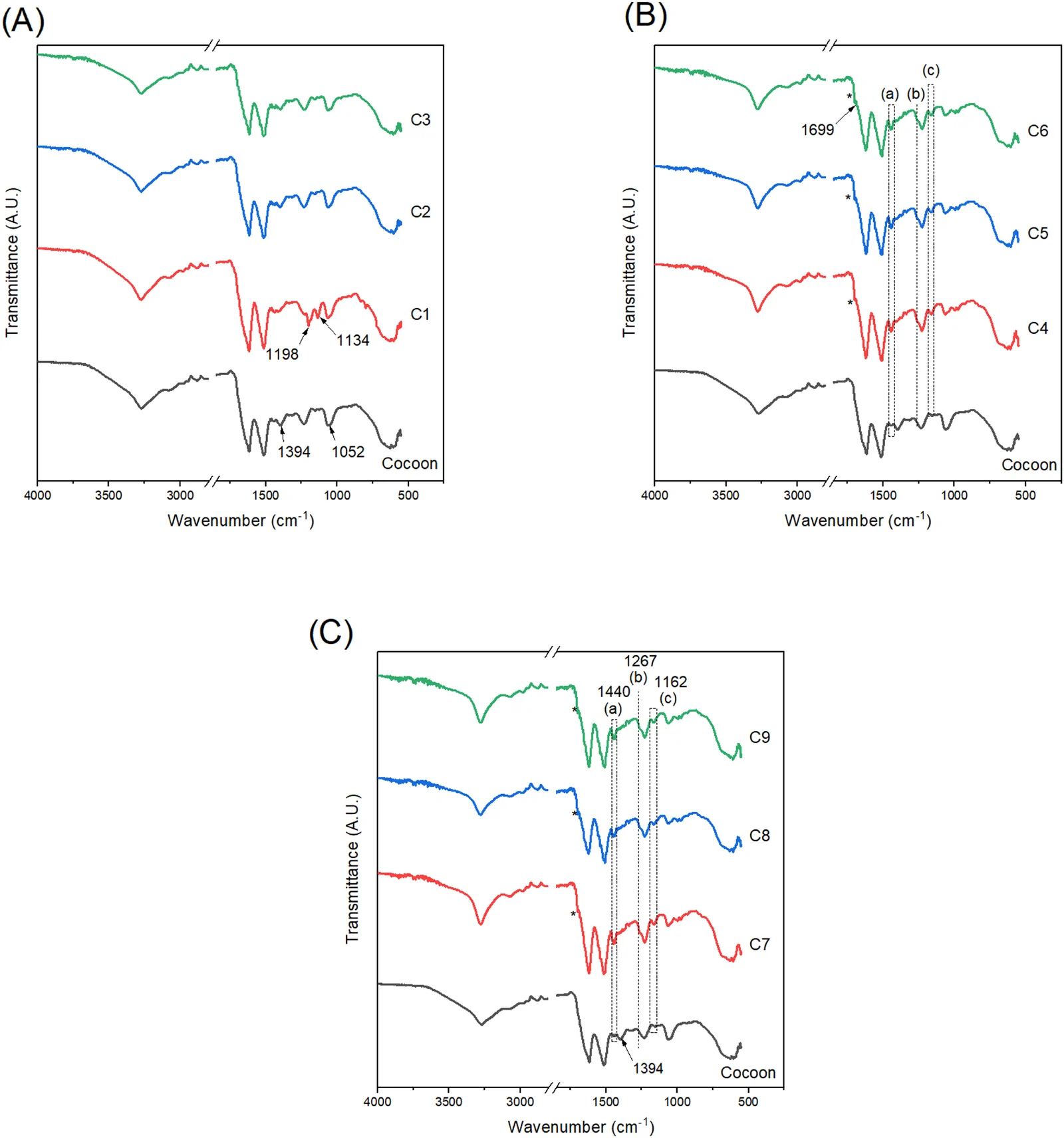
FTIR-ATR spectra of native silkworm cocoon fibers and RSF. (A) Boiling water C1, 0.25% SLES C2, and 0.5% SLES C3. (B) 0.25% Na_2_CO_3_ C4, 0.25% Na_2_CO_3_ + 0.25% SLES C5, and 0.25% Na_2_CO_3_ + 0.5% SLES C6. (C) 0.5% Na_2_CO_3_ C7, 0.5% Na_2_CO_3_ + 0.25% SLES C8, and 0.5% Na_2_CO_3_ + 0.5% SLES C9. The cocoon references are in the bottom graph in Figures A, B, and C.

Figure 3A shows a vibrational mode associated with C=O stretching (Amide I) at 1615 cm^–1^ (silk cocoon, C1 and C3) and at 1617 cm^–1^ (C2). Figure 3B C4 and C6 exhibit Amide I at 1620 cm^–1^, meanwhile C5 at 1619 cm^–1^. And in Figure 3C, Amide I is present at 1619, 1620, and 1617 cm^–1^ (C6, C7, and C9, respectively). All correspond to a β-sheet conformation.^33^

Amide II is observed at 1509 cm^–1^ (C6, C8, and C9), 1510 cm^–1^ (C1), 1511 cm^–1^ (C4 and C5), 1513 cm^–1^ (cocoon and C2), and at 1515 cm^–1^ (C3 and C7), corresponding to N–H bending.

Amide III is presented at 1224 cm^–1^ (C3), 1227 cm^–1^ (C4, C5, C8, and C9), 1229 cm^–1^ (C6 and C7), 1230 cm^–1^ (raw cocoon and C1), and 1231 cm^–1^ (C2) corresponding to C–N stretching and N–H bending modes.

Spectra for degummed silks in Figure 3B displayed two absorption bands at 1440 cm^–1^ (a) and 1162 cm^–1^ (c), related to asymmetric bending modes of CH_3_ and N–C_α_ stretching, respectively,^34^ with an increase in intensity from C4 to C6, and from C7 to C9 for the spectrum in Figure 3C. Meanwhile, for degummed silks in Figure 3A, the intensity of these absorption bands is barely noticeable. A displacement of the N–C_α_ stretching of C1 from 1198 to 1134 cm^–1^ is observed. On the other hand, the degummed silks also exhibit a low-intensity peak at 973 cm^–1^, attributed to a polyalanine glycine (AG)_n_ β-sheet structure. At 1699 cm^–1^, a slight peak (indicated by an asterisk) is observed for degummed C4 to C9; this absorption band is associated with a β-sheet conformation and serves as an indicator of protein crystallinity.^35^

Also, from degummed C4 to C9 FTIR spectra, a shoulder around 1265–1269 cm^–1^ (b) is observed, which is attributed to the random coil conformation of silk fibroin that was hidden by the sericin protein. This was not observed in sample C1 (where only water was used) or in samples C2 and C3 (where SLES was used as the degumming agent). In addition, it is observed a decrease in the intensity band of all the degummed processes except for cocoon, C1, C2, and C3 at 1052 cm^–1^ (C–C bending vibration), attributed to the sericin presence, and the disappearance of the band at 1394 cm^–1^ (C–H bending vibration),^34^ from sample C4 onward, as an indication of the absence of sericin protein. Based on the above, the successful removal of sericin from degummed samples can be achieved via alkaline treatment. A summary of the positions and assignments of the characteristic bands is described in Table 3.

**Table 3:** Summary of the Main Vibrational Modes of the Silkworm Cocoon and the Degummed Silkworm Cocoon

Frequency (cm^–1^)	Vibrational mode	Conformation	References
3275	N–H stretching (Amide A)	-	^36^
3070	N–H stretching (Amide B)	-	^36^
1699	C=O stretching (Amide I)	β-sheets/β-turns	^34^
1615–1620	C=O stretching (Amide I)	β-sheet	^33^,^37,38^
1509–1515	N–H bending (Amide II)	β-sheet	^32^,^33^
1439–1440	Asymmetric bending CH_3_	β-sheet	^34^,^35^
1265–1269	C–N stretching and NH bending (Amide III)	α-helices	^32^
1230	C–N stretching and NH bending (Amide III)	β-sheet	^32^,^33,39^
1162	N–C_α_ stretching	Distribution of conformation	^34^
1052	C–C bending	β-sheet structure from sericin	^34^
973	r(CH_2_), (AG)_n_	β-sheet	^35^

### 3.3 Chemical Composition by NMR

The NMR spectrum of the RSF is shown in Figure 4. At 29.41 and 29.63 ppm, corresponding to C2 and C3 degummed silk cocoon, respectively, a peak appears corresponding to the Glu C_γ_ amino acid residue,^40–42^ in both degummed and processed with SLES. The same phenomenon is observed in C4 and C7 degumming, with peaks at 32.2 and 32.39 ppm, respectively. This peak is associated with the presence of the amino acid Tyr C_β_.^43^ In this case, these degummed samples were processed with Na_2_CO_3_. Another degumming process that demonstrates the contribution of the peaks described above is degumming C5, processed with a combination of degumming agents at 0.25% w/v each of Na_2_CO_3_ and SLES, yielding 32.38 and 29.47 ppm, respectively. The main chemical shift attributed to the degummed silk of *B. mori* is described in Table 4.

**Figure 4:**
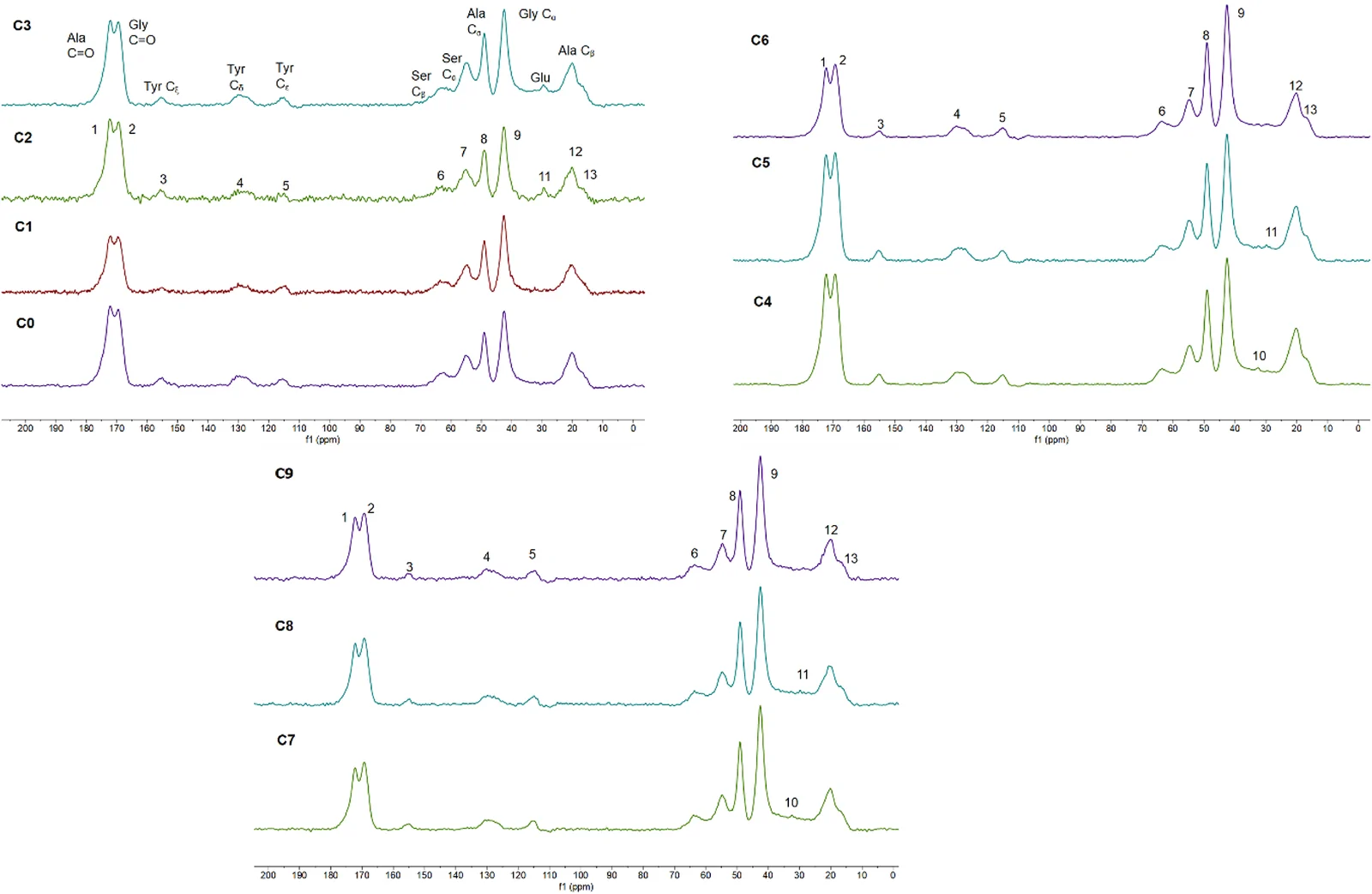
^13^C NMR spectrum of silkworm cocoon compared to degummed samples.

**Table 4:** ^13^C-NMR Chemical Shift of Main Amino Acid Residues in Degummed Silk Structure

^13^C labeling	Chemical shift (ppm)	Amino acid	Carbon
1	172.39	Ala	C=O
2	169.15	Gly	C=O
3	155.14	Tyr	C_ξ_
4	129.39	Tyr	C_δ_
5	115.21	Tyr	C_ε_
6	63.23	Ser	C_β_
7	55	Ser/(Tyr)	C_α_
8	49.11	Ala	C_α_
9	42.52	Gly	C_α_
10	32	Tyr	C_β_
11	29	Glu	Cγ
12	20.28	Ala	C_β_
13	16	Ala	C_β_

### 3.4 Membrane Characterization

The solution concerning E2 was very viscous and failed to form a Taylor cone, thus preventing the formation of fibers and, therefore, the manufacture of the E2 membrane.

#### 3.4.1 Morphology and Surface Analysis

The morphology, uniformity, and fiber diameter of the electrospun membranes were analyzed using SEM. The morphological behavior is characteristic of each group, namely, electrospun membranes with 3% w/v and 5% w/v fibroin, respectively.

As shown in Figure 5a and Figure S4, the membranes with 3% w/v fibroin (E1, E3, E5, E7, E9, E11, E13, and E15) show less dispersion in fiber diameter. Two of them have average diameters of 0.374 and 0.390 μm, respectively, and the other six membranes have diameters between 0.5 and 0.6 μm. Electrospun membrane E11 shows the greatest homogeneity in fiber diameter (Figure S4, h), whereas E15 is the most heterogeneous (Figure S4, k). Furthermore, membranes E5 and E13 share features in morphology and diameter size distribution.

**Figure 5:**
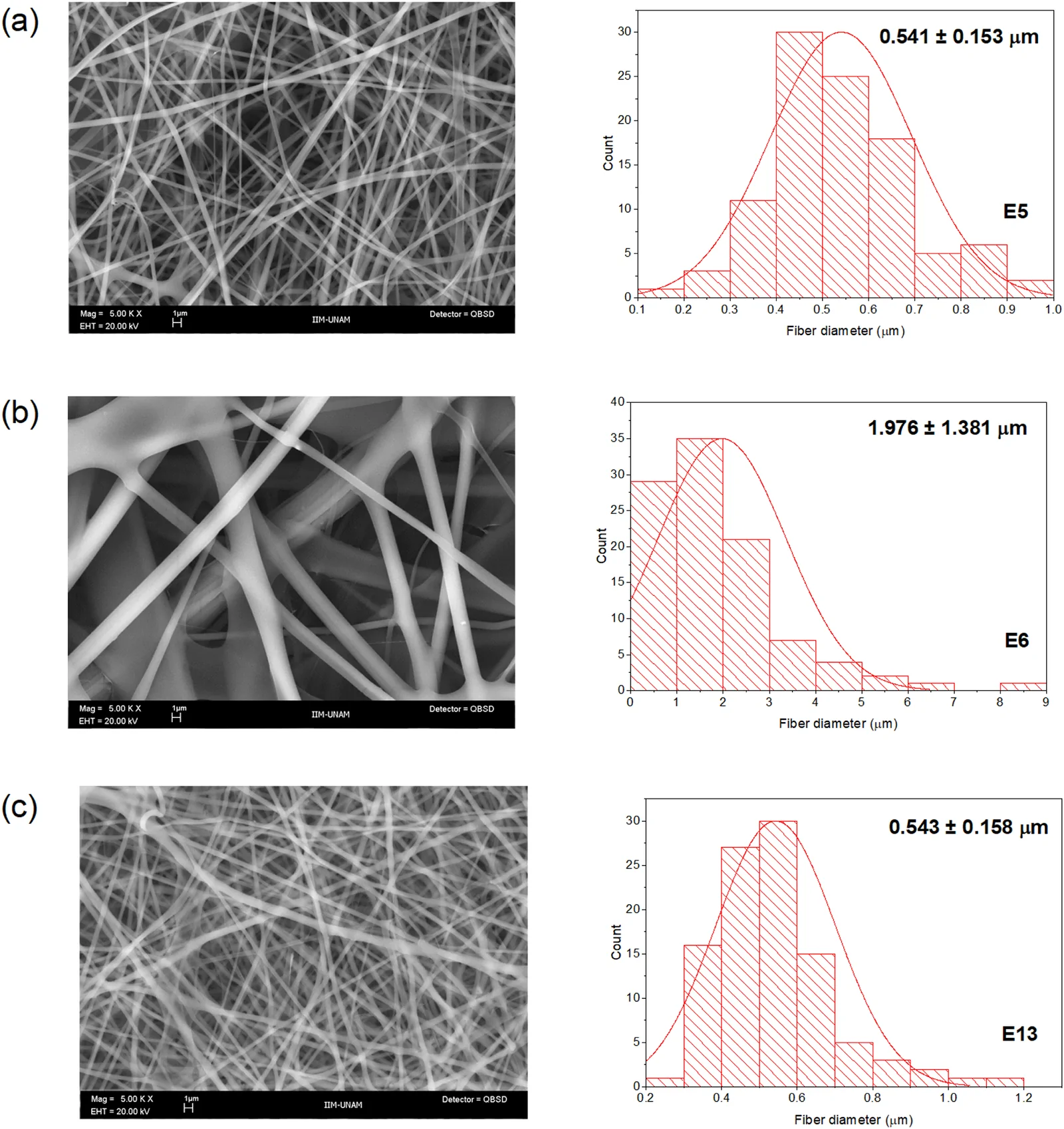
Distribution of the average fiber diameter obtained by electrospinning and its respective micrograph. Micrographs at 5000× magnification. In the figure, the morphology and fiber diameter distribution of electrospun membranes E5 (a), E6 (b), and E13 (c) are shown.

Meanwhile, for electrospun membranes manufactured at 5% w/v fibroin concentration (E4, E6, E8, E10, E12, E14, and E6), the diameter size distribution is above 0.6 μm. There is a large deviation in all cases, with a maximum of 1.381 μm for membrane E6 (Figure 5b).

There is an increase in fiber when high flow rates (even samples) are applied, due to incomplete solvent evaporation before the sample reaches the collector.

In Figure 6, as expected, differences are observed in the fibroin layers deposited randomly during the electrospinning. The average surface roughness (Sa) and mean ± SD values of each electrospun membrane are presented. All the electrospun membranes differed significantly from the E4 membrane (12.73 ± 2.07 μm).

**Figure 6:**
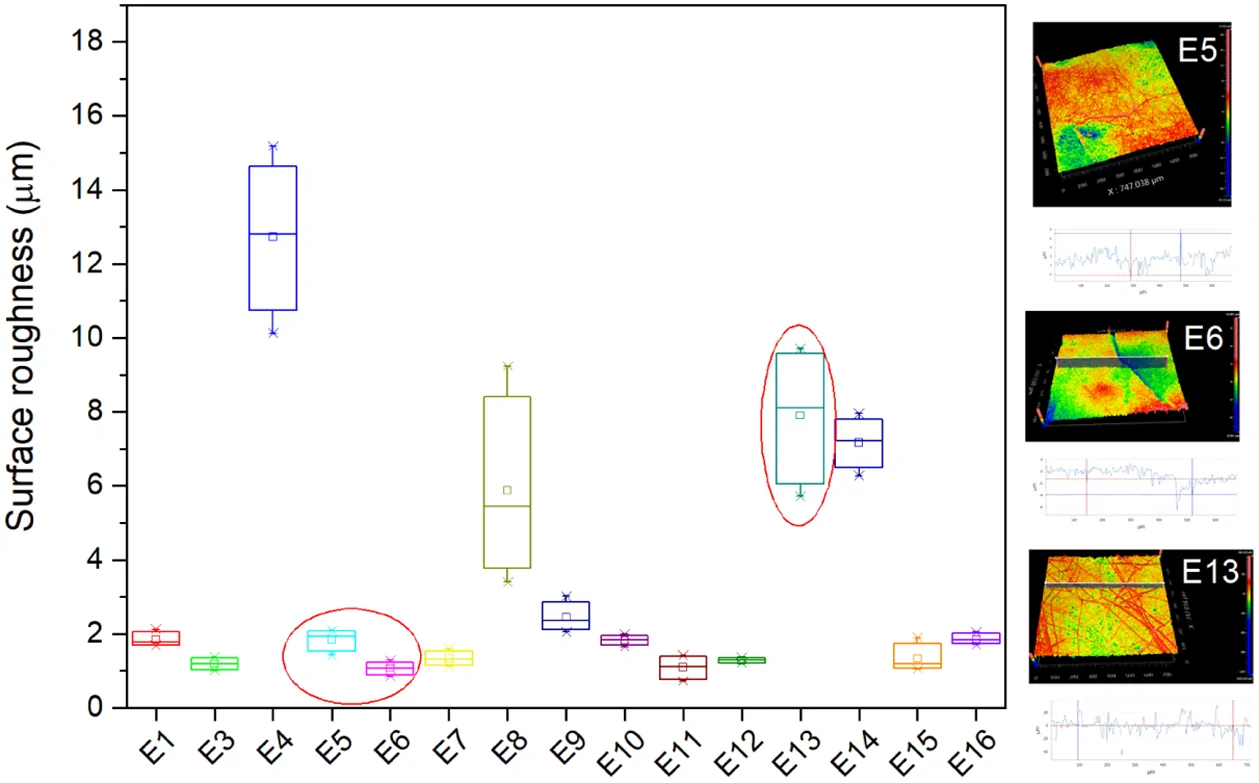
Graphical representation of the average roughness surface of the electrospun membranes measured on an area of approximately 750 μm^2^ by optical profilometry.

Those with the best biological response are circled, and the surface topography and profiles are shown. Membranes E5 and E6 showed similarity in surface roughness, with values of 1.84 ± 0.29 μm and 1.06 ± 0.18 μm, respectively. Furthermore, flaws were observed in membranes E5 (holes) and E6 (grooves). Despite these topographic defects in the membranes, membrane E13 has a roughness of 7.9 ± 1.84 μm, due to greater variation between peaks and valleys (surface irregularities).

For the remaining electrospun membranes, the estimated roughness in the profilometer images (Figure S5) matches that observed in the SEM micrographs.

Electrospun membranes with an appropriate biological response exhibited contact angles greater than 90°, indicating hydrophobic behavior (Figure 7). Being E5 the sample with the lowest contact angle (91.8° ± 5.9), followed by E6 (101.5° ± 2.1), and with the highest contact angle E13 (122.1° ± 3.2). The descending order of hydrophilicity corresponds to the cellular response, showing that E5 is the sample that promotes functional cell morphology, while E13 promotes cell aggregation into clusters as a result of the surface’s low wettability.

**Figure 7:**
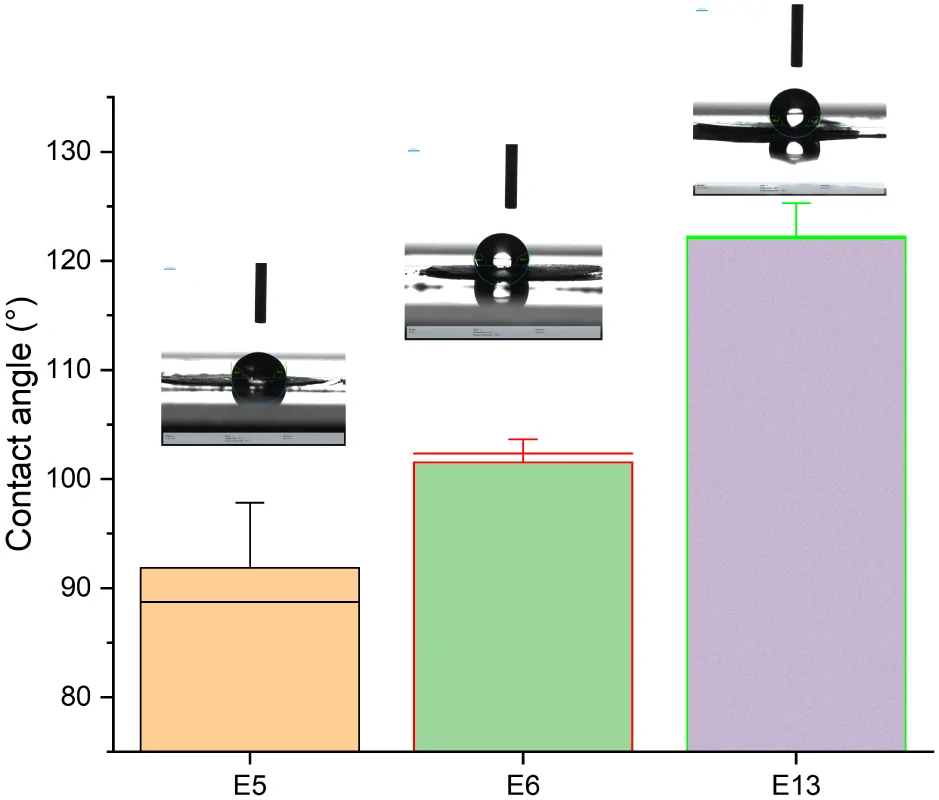
Wettability surface of electrospun membranes. Hydrophobic behavior is observed in ascending order (E13 > E > E5).

Despite this initial hydrophobic behavior, the contact angle decreases within seconds, allowing for close cell-material interaction.

On the other hand, Figure 8 shows the changes in dimensions due to exposure to wet conditions at physiological temperature after 72 h of study. The electrospun membrane E5 exhibits a notable decrease in its dimensions, with a 47% decrease in area. Sample E6 exhibits similar behavior but with a smoother slope; dimensions remained stable between days 1 and 2, then shrank on the third day by 28% in area. Meanwhile, E13 shows dimensional stability and a slight increase, with a 4.5% area loss. This result is consistent with studies on contact angle, in which E13 exhibits the largest contact angle and cellular behavior.

**Figure 8:**
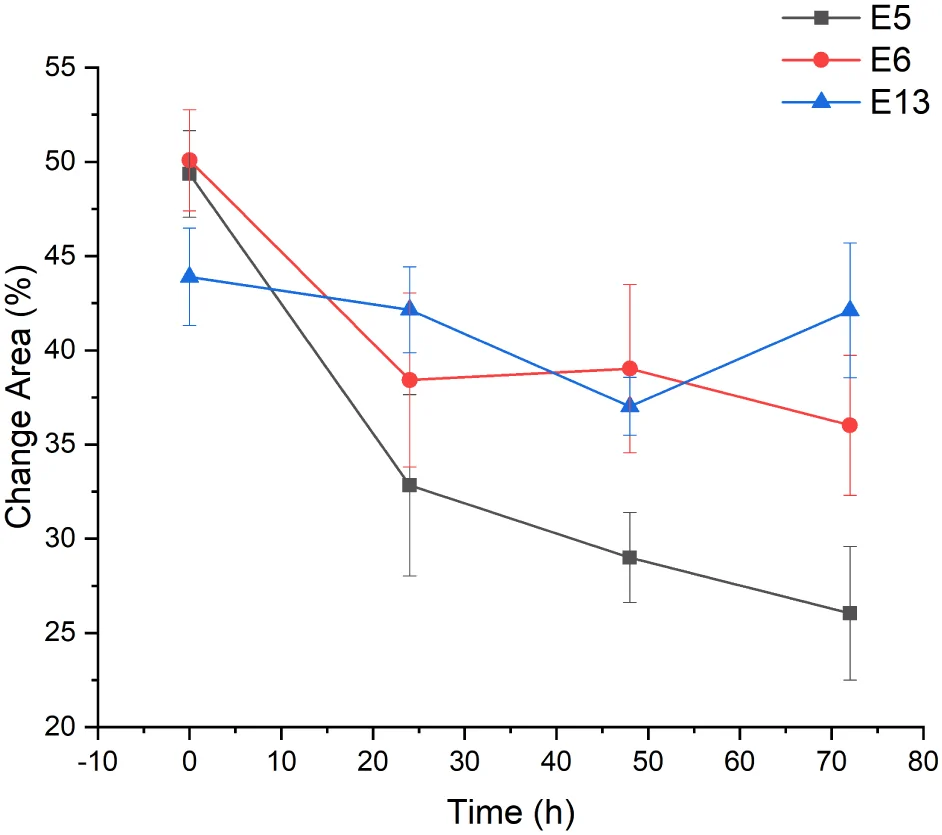
Graph of area change for E5, E6, and E13 electrospun membranes at 0, 24, 48, and 72 h immersed in distilled water at 37 °C.

#### 3.4.2 Chemical Composition by FTIR

The FTIR-ATR spectra of the RSF electrospun membranes are shown in Figure 9. The silk cocoon fibers exhibit high-intensity bands at 1629 cm^–1^, 1518 cm^–1^, and 1231 cm^–1^. The first two correspond to secondary beta-sheet structures, and the last to a random coil conformation.

**Figure 9:**
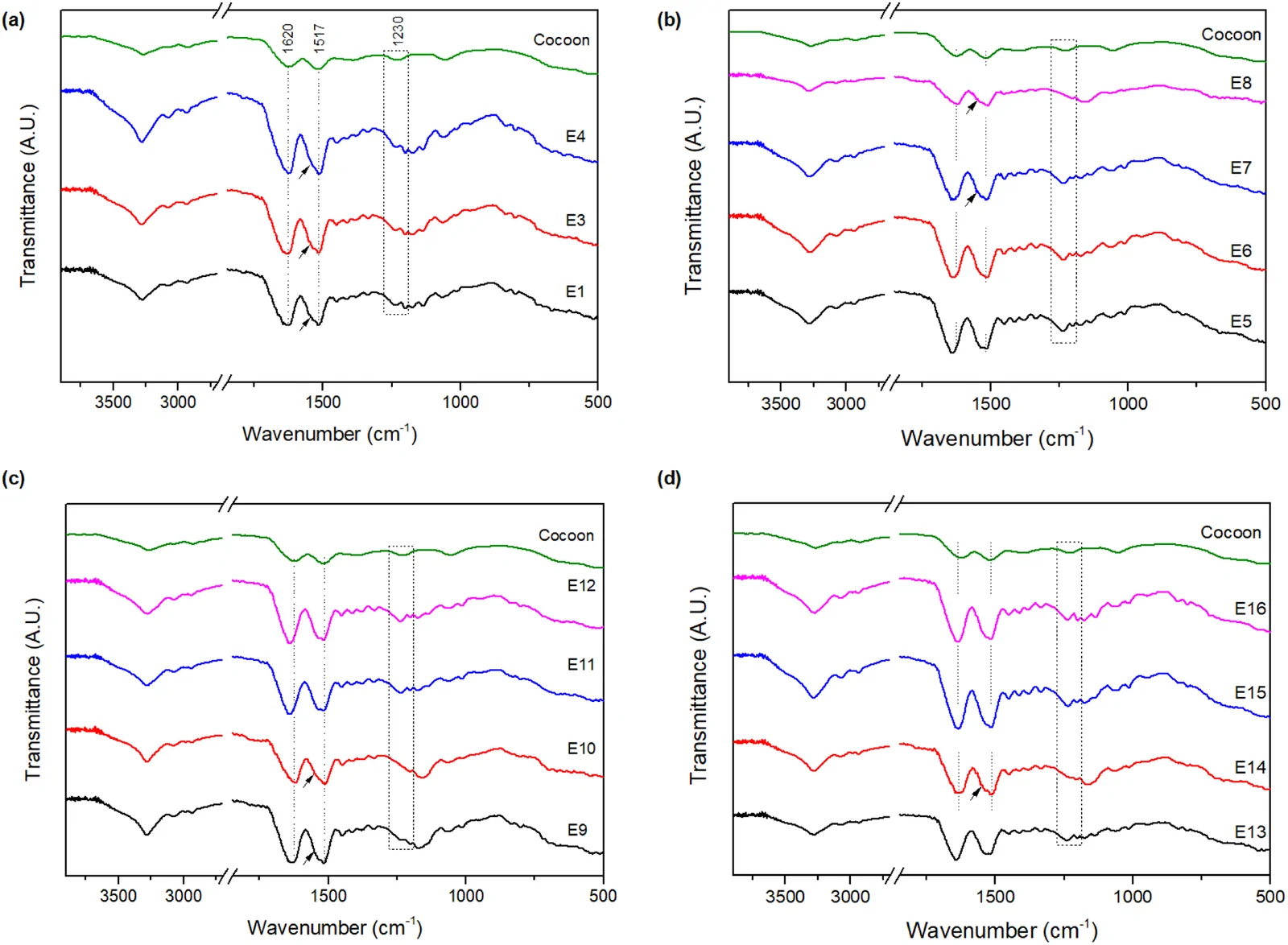
FTIR spectra of electrospun nanofibers. Silkworm cocoon spectrum is included. FTIR spectra of electrospun membranes (a) E1, E3, and E4; (b) E5, E6, E7, E8, (c) E9, E10, E11, E12, and (d) E13, E14, E15, E16.

All electrospun RSF exhibit a shoulder at 1540 cm^–1^ associated with a random conformation and α helix structures due to Amide II and attributed to silk I conformation. And a band splitting of the secondary structure of Amide III at 1235 cm^–1^ and 1200 cm^–1^. The bands at 1620 and 1516 cm^–1^ displayed strong intensities, indicating an increase in β-sheet structure. This may occur because the solvent evaporates rapidly during electrospinning, particularly along the path from the needle to the collector, thereby preventing the fibroin chains from settling and partially inhibiting the formation of a crystalline phase.

#### 3.4.3 Crystallinity by XRD

Figure 10 shows the X-ray diffraction patterns of the silkworm cocoon and the RSF electrospun membranes. All samples exhibit a diffraction peak around 21°, due to the contribution of β-sheets (silk II) structure. A narrow peak with greater intensity is observed in the silkworm cocoon, unlike the electrospun membranes, where lower intensity and a broader peak are observed, suggesting the presence of α-helices (silk I) around 25°. This infers an amorphous contribution, which is reflected with a decrease in the degree of crystallinity. This may be due to the interaction of TFA, which inhibits the molecular organization of secondary structures.^44^

**Figure 10:**
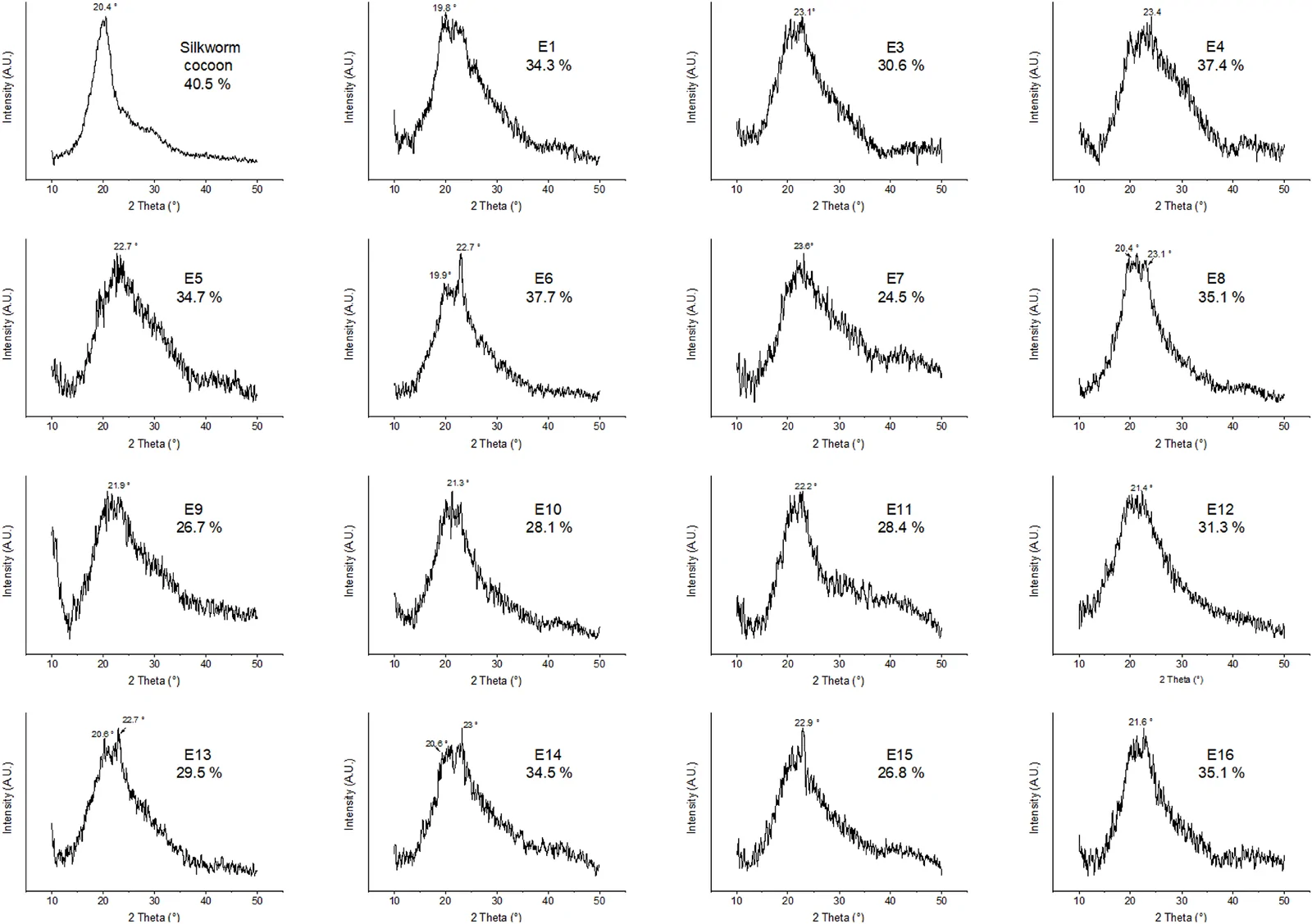
DRX pattern of native silkworm cocoon fibers vs electrospun membranes of silk fibroin. For each sample, the percentage of crystallinity is described.

#### 3.4.4 Mechanical Properties

Based on in vitro analysis, membranes E5, E6, and E13 preserved the elongated morphology characteristic of fibroblasts. Therefore, based on this finding, only mechanical studies were performed on these samples.

As shown in Figure 11, the mean value and standard deviation of the hardness (a) and elastic modulus (b) were calculated using the Oliver-Pharr method. Electrospun membrane E13 shows the greatest deformation in the microindentation test, with a penetration depth of 200–250 μm, compared to E5 and E6, which both showed similar behavior (70–120 μm) (Figure S6). The main difference in Young’s modulus is described by the slope of the unloading curve, defined as the contact stiffness (S). Electrospun membranes display a local modulus of elasticity of 263.1 ± 91 (E5), 389.2 ± 168 (E6), and 49.6 ± 4 (E13) MPa.

**Figure 11:**
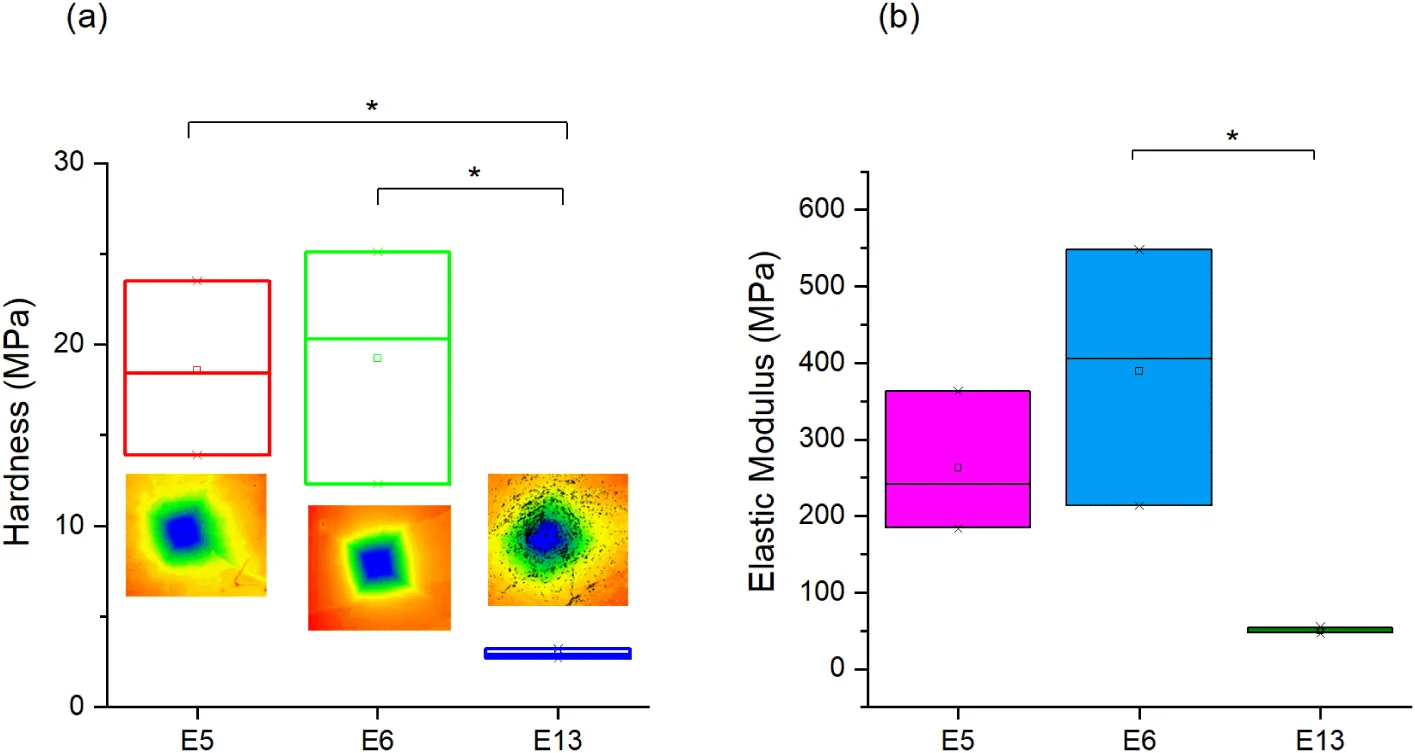
Microindentation results. (a) Hardness where an indentation imprint is shown, and (b) elastic modulus of electrospun membranes. Statistical analysis was performed using one-way ANOVA with Tukey test (*p < 0.05).

Hardness may be associated with the definition of the margins of the indentation imprint on the sample. As shown in Figure 11a, scaffold E13 (2.94 ± 0.3 MPa) has the most diffuse indentation imprint, followed by scaffold E5 (18.6 ± 4.8 MPa) and membrane E6 (19.23 ± 6.5 MPa), which has the best resolution.

These outcomes suggest that C4 degumming conditions positively affect the biological response, particularly in preserving cell morphology.

### 3.5 Cell Viability and Cytotoxicity

Cytocompatibility assays demonstrated a concentration-dependent response. Alkaline-treated membranes maintained viability above 80%, whereas surfactant-only treatments showed reduced metabolic activity at full extract concentration. A structure–cytocompatibility relationship was identified, suggesting that increased crystallinity and effective sericin removal reduce the release of soluble residues and improve biological response.

The indirect cytotoxicity of electrospun silk fibroin membranes obtained under different degumming conditions (C2–C9) was evaluated using the MTT assay after 24 h exposure of human dermal fibroblasts (ATCC PCS-201-012) to scaffold extracts at 100%, 75%, and 50% concentrations (Figure 12).

**Figure 12:**
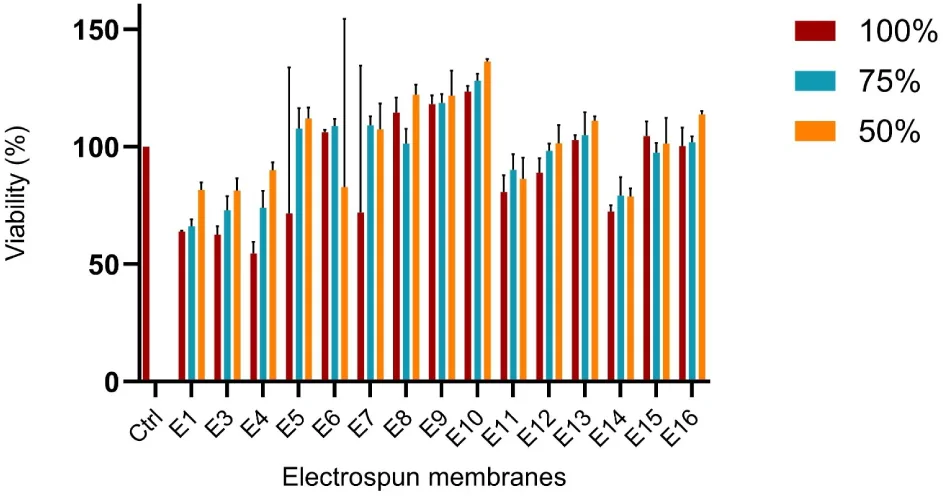
Cell viability assay of electrospun membranes after 24 h incubation with silk fibroin liquid extracts.

At 100% extract concentration, membranes derived from condition C2 (E1) and C3 (E3–E4) exhibited the lowest cell viability values (approximately 55–70%). In contrast, membranes from conditions C4–C9 maintained viability levels above 80%, with several groups exceeding 100%, indicating preserved or enhanced metabolic activity.

The observed dose-dependent response suggests that residual soluble compounds released from inadequately degummed fibroin may transiently affect mitochondrial activity. Alkaline degumming appears to reduce such effects by more effectively eliminating sericin and residual surfactant.

A concentration-dependent trend was observed across most samples, with decreasing extract concentration from 100% to 50% increasing cell viability. At 50% extract concentration, all evaluated membranes exhibited viability values above the 70% cytocompatibility threshold.

These findings indicate that the biological response of fibroblasts is influenced by both degumming parameters and the concentration of soluble compounds released from the electrospun membranes.

According to the results of the calcein assay shown in Figure S7, the typical morphology of the fibroblasts was not preserved. However, for membranes E5, E6, and E13 (cell clusters formation), preserved elongated fibroblast morphology is observed in calcein assays (Figure 13), indicating favorable cell–material interactions. These samples were therefore selected for mechanical testing, aligning structural optimization with biological performance.

**Figure 13:**
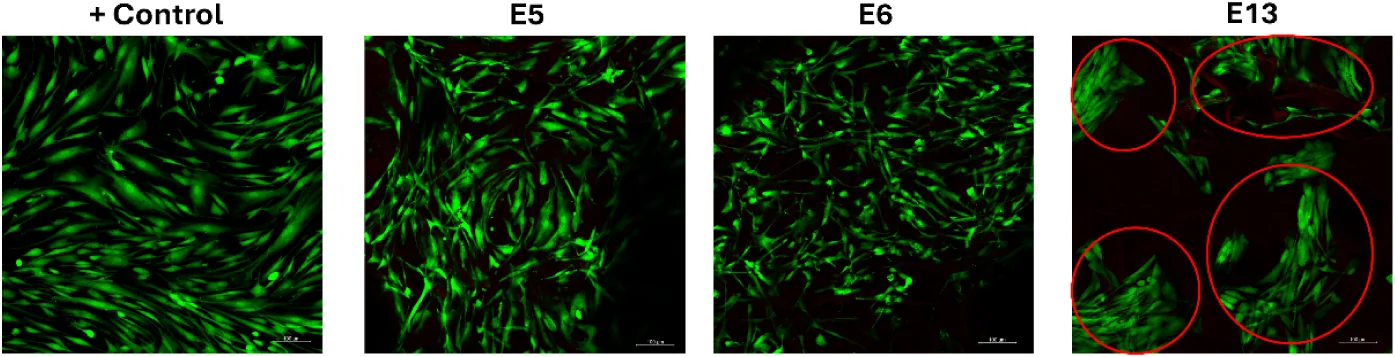
Micrographs of calcein tests. Bar scale at 100 μm. In red circles are appreciated cell clusters.

The structural characterization results provide mechanistic insight into the biological response observed in the cytocompatibility assays. Alkaline-based degumming treatments (C4–C9), which achieved higher sericin removal efficiency and promoted β-sheet exposure, also yielded electrospun membranes with relatively high crystallinity (up to ∼37%). In contrast, surfactant-only treatments (C2–C3), associated with incomplete sericin removal and weaker β-sheet signals in FTIR spectra, correspond to a low degree of crystallinity and reduced cell viability at full extract concentration.

This relationship suggests that effective sericin elimination not only purifies fibroin but also stabilizes its secondary structure, reducing the release of residual soluble compounds during extract preparation. Since the MTT assay reflects mitochondrial metabolic activity, the improved viability observed in membranes derived from alkaline treatments likely indicates a more chemically stable, biologically inert surface.

Moreover, higher crystalline content may limit molecular mobility and decrease the diffusion of low-molecular-weight residues into the culture medium, thereby attenuating dose-dependent metabolic interference. These findings indicate that modulation of crystallinity and secondary structure may act as an indirect regulator of cytocompatibility in electrospun silk fibroin membranes.

Recent studies have shown that optimization of polymer processing and electrospinning parameters can substantially improve the functional properties of electrospun membranes by modulating their structural organization. For example, Alkhaldi et al. 2026 ^45^ reported that electrospun chitosan/poly­(vinyl alcohol) membranes exhibited enhanced mechanical strength, hydrophilicity, and antifouling performance as a consequence of improved intermolecular interactions and optimized structural organization. Although their work was directed toward membrane separation rather than tissue engineering, it similarly demonstrates that careful control of processing parameters is essential for tailoring structure–property relationships in electrospun biopolymer systems.

Comparable observations have been reported for electrospun silk fibroin biomaterials. Recent reviews emphasize that degumming conditions, solvent selection, electrospinning parameters, and postprocessing treatments collectively influence fibroin secondary structure, fiber morphology, crystallinity, mechanical properties, degradation behavior, and ultimately the biological performance of the resulting scaffolds.^9^ Likewise, optimization of silk fibroin fibrous architecture has been shown to improve scaffold stability and cellular interactions, supporting enhanced tissue regeneration and wound healing.^46^ Furthermore, studies evaluating silk fibroin-based electrospun membranes have demonstrated that processing-induced structural modifications affect degradation profiles and mechanical integrity, both of which are closely associated with the biological performance required for regenerative medicine applications.^47^

Collectively, these studies, together with our findings, support the concept that degumming should be regarded as a critical upstream processing step whose optimization is associated with the structural organization and subsequent cytocompatibility of electrospun silk fibroin membranes. Although additional mechanistic studies are required to determine the specific contributions of crystallinity and other structural features to cellular behavior, the present work demonstrates that systematic control of degumming conditions provides a practical strategy for producing electrospun silk fibroin membranes with reproducible structural characteristics and favorable cytocompatibility profiles suitable for tissue-engineering applications.

### 3.6 Antibacterial Activity Test

The antibacterial performance of electrospun fibroin membranes was evaluated against *Staphylococcus aureus* ATCC 25923 and *Pseudomonas aeruginosa* ATCC 27853 (Figure 14). Optical density measurements after 18 h incubation showed no statistically significant reduction in bacterial growth compared to positive controls.

**Figure 14:**
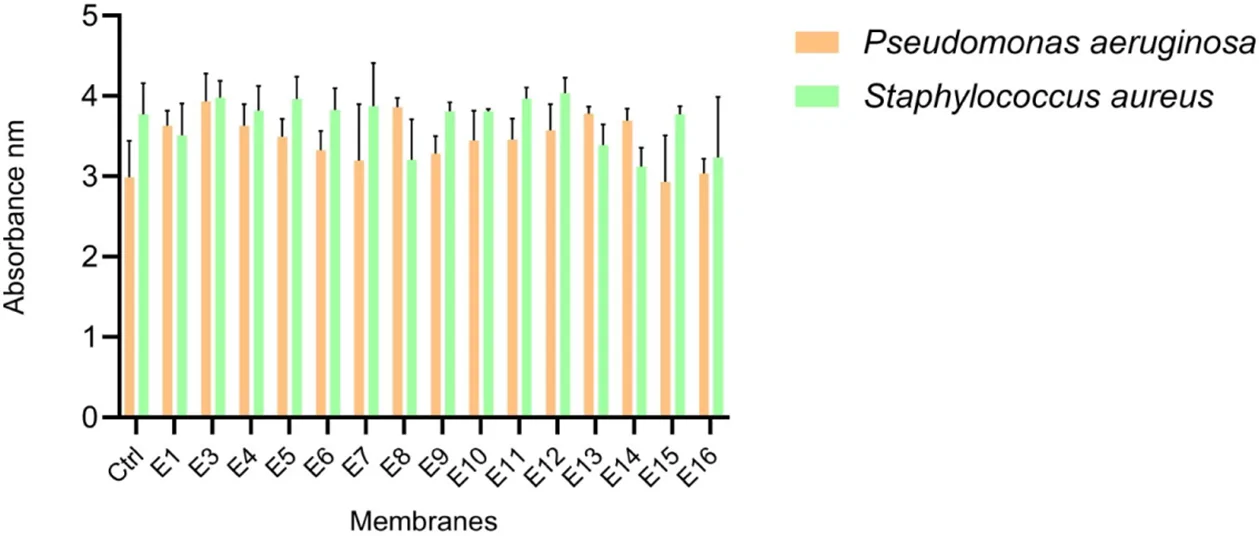
Antibacterial assay of electrospun fibroin/sericin membranes against *Staphylococcus aureus* and *Pseudomonas aeruginosa* after 18 h of incubation. Ctrl shows positive control.

These results indicate that the degumming conditions and subsequent electrospinning process do not confer intrinsic antibacterial properties to the scaffolds. This outcome is consistent with the chemical composition of purified silk fibroin, which lacks inherent antimicrobial functional groups.

Importantly, the absence of antibacterial activity confirms that no unintended antimicrobial effects were introduced during chemical processing. For applications requiring infection control, additional functionalization strategies—such as—incorporation of bioactive agents or surface modification—would be necessary.

## 4 Conclusions

This study demonstrates that the degumming strategy is a decisive upstream parameter governing the structural organization and biological performance of electrospun silk fibroin membranes. Through a controlled experimental design combining alkaline (Na_2_CO_3_) and surfactant (SLES) treatments, it was shown that alkaline-based degumming significantly enhances sericin removal efficiency, promotes β-sheet exposure, and modulates crystallinity in regenerated fibroin.

Chemical and structural characterization by mass loss, FTIR-ATR, solid-state ^13^C NMR, and X-ray diffraction confirmed that effective alkaline degumming improved fibroin purification and more stable secondary structural domains. These structural differences were directly reflected in the cytocompatibility response. Membranes derived from alkaline-treated fibroin exhibited improved morphology, mechanical properties, and metabolic activity while preserving fibroblast morphology. In contrast, surfactant-only treatments showed reduced viability at higher extract concentrations.

A structure–cytocompatibility relationship was identified, suggesting that increased crystallinity and effective sericin elimination may contribute to improved early cellular metabolic activity. Although the scaffolds lacked intrinsic antibacterial activity, they exhibited favorable cytocompatibility profiles suitable for tissue engineering applications.

The mechanical properties determined by the microindentation test proved useful for fibroin nanofibers and, to our knowledge, are first reported for it and many other polymer nanofibers. Mechanical studies of electrospun membranes show that intermediate hardness and stiffness values, such as those observed in E5, promote an elongated morphology with fluorescence intensity similar to that of the control. In contrast, a stiffer, harder membrane, such as E6, alters the typical morphology of fibroblasts, whereas a “soft” electrospun membrane (E13) leads to the accumulation of cells in clusters. Based on these results, the electrospun membrane E5 provides a microenvironment that enables cells to maintain appropriate morphology, thereby preserving cellular functions. This last one is also regulated by surface roughness and wettability, which promote fibroblast spreading and, therefore, play a role in cell migration and, consequently, in tissue regeneration or wound healing. In the dimensional retention test, E13 maintained morphological stability for at least 72 h, suggesting its potential for medical applications.

Overall, this work highlights degumming as a key structure-controlling step in the fabrication of electrospun silk fibroin membranes, with structural modifications being associated with differences in cytocompatibility. These findings provide useful guidance for optimizing processing conditions during the development of silk-based scaffolds for tissue engineering applications.

## Supplementary Material



## Notes

Statistical design of experiment (DOE); Taguchi analysis of electrospinning conditions; raw data processing of the FTIR spectrum in Origin software; morphology of the micrographs of the degumming process; distribution of the average fiber diameter of electrospun membranes; surface roughness of electrospun membranes; microindentation curves for triplicate electrospun membranes; micrographs of calcein assay for membranes without preserving elongated morphology (PDF)

## #

The co-author, Stephen Muhl Saunders, passed away on March 27th, 2026.
