# Supplementary material for: Process-Driven Modulation of Structure and Cytocompatibility in Electrospun Silk Fibroin Membranes through Controlled Degumming
Source: ACS Omega. 2026 Aug 22;11(35):52928–42. doi: 10.1021/acsomega.6c04899 (PMC13563627; doi:10.1021/acsomega.6c04899)
Supplement: Supplementary file 1 [file ao6c04899_si_001.pdf]

SUPPLEMENTARY MATERIAL

Process-driven modulation of structure and cytocompatibility in electrospun silk fibroin membranes through controlled degumming

K.K. Gómez-Lizárraga[1], E. Robles-Hurtado[2], H.A. Méndez-Escobar[3], C. Velasquillo[3*], Stephen Muhl Saunders[2] ł, J. Cruz-Cárdenas[4,2], R. Vera-Graziano[2*]

[1] Programa Investigadoras e Investigadores por México. Secretaría de Ciencia, Humanidades, Tecnología e Innovación / Instituto de Investigaciones en Materiales, Universidad Nacional Autónoma de México, Circuito Exterior S/N. Circuito de la Investigación Científica, Ciudad Universitaria, 04510, Ciudad de México, México.
[2] Instituto de Investigaciones en Materiales, Universidad Nacional Autónoma de México, Circuito Exterior S/N. Circuito de la Investigación Científica, Ciudad Universitaria, 04510, Ciudad de México, México.
[3] Unidad de Ingeniería de Tejidos, Terapia Celular y Medicina Regenerativa, Instituto Nacional de Rehabilitación Luis Guillermo Ibarra Ibarra, 14389, Ciudad de México, México.
[4] Facultad de Ciencias, Universidad Nacional Autónoma de México, 04510 Ciudad de México, México.

*Corresponding authors

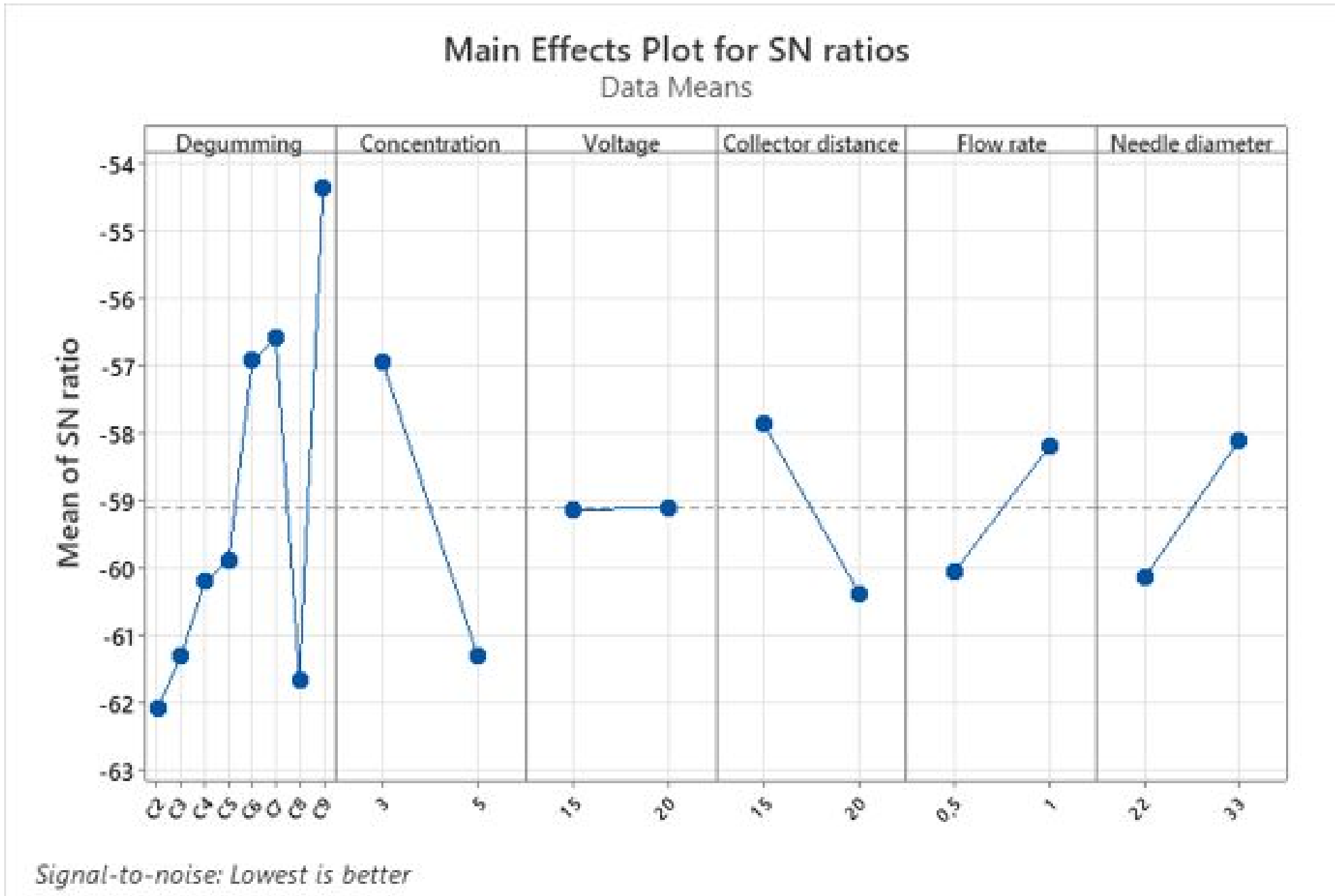

Figure S1. Statistical design of experiment (DOE) Taguchi analysis of electrospinning conditions.

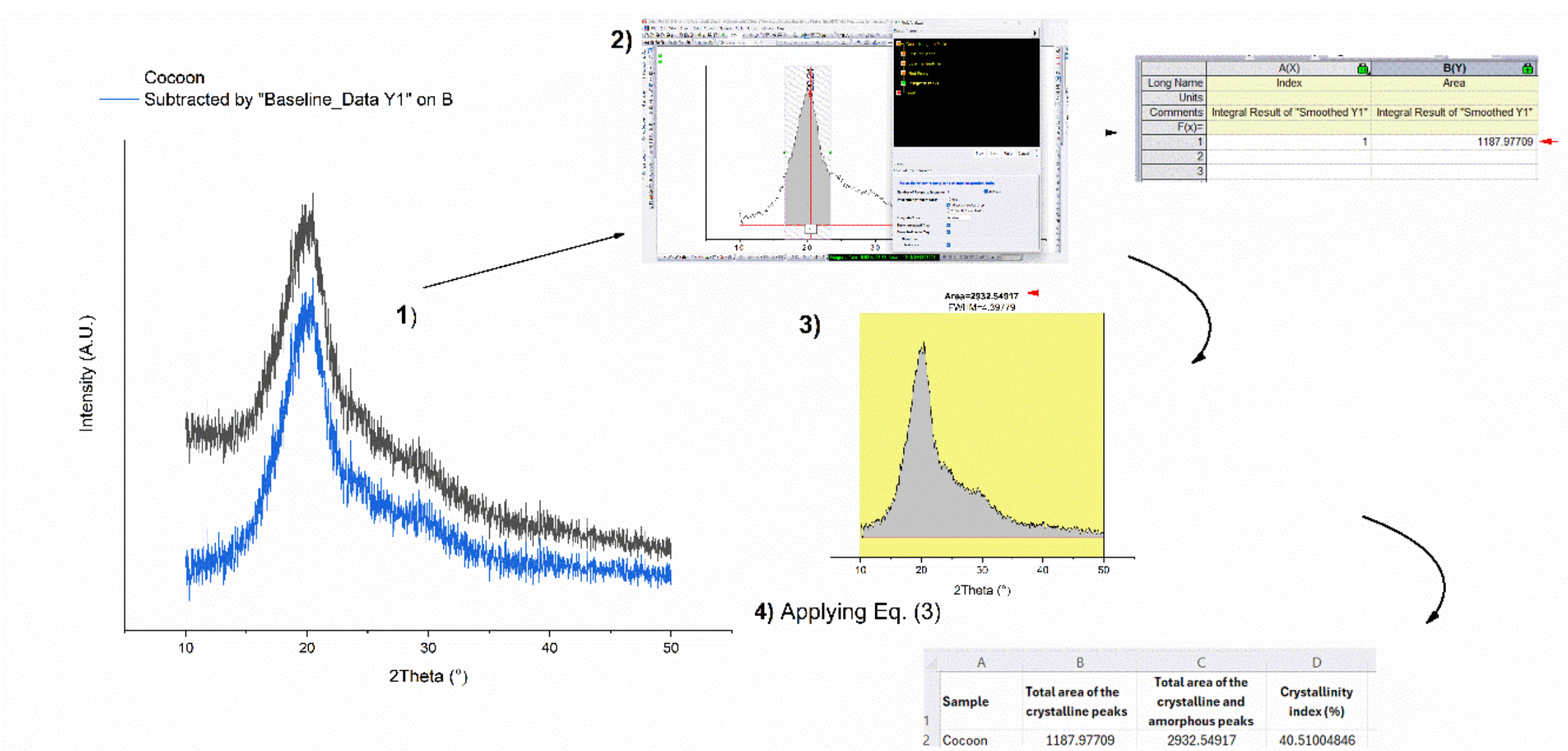

Figure S2. Raw data processing in Origin software. 1) Peak selection and subtraction of the baseline, 2) determination of the area under the curve (crystalline region) based on its processing with the Peak Analyzer tool, 3) determination of the total area of the diffractogram, which considers the contributions of the amorphous and crystalline regions, and 4) calculation of the crystallinity index from the determined areas using Equation 3.

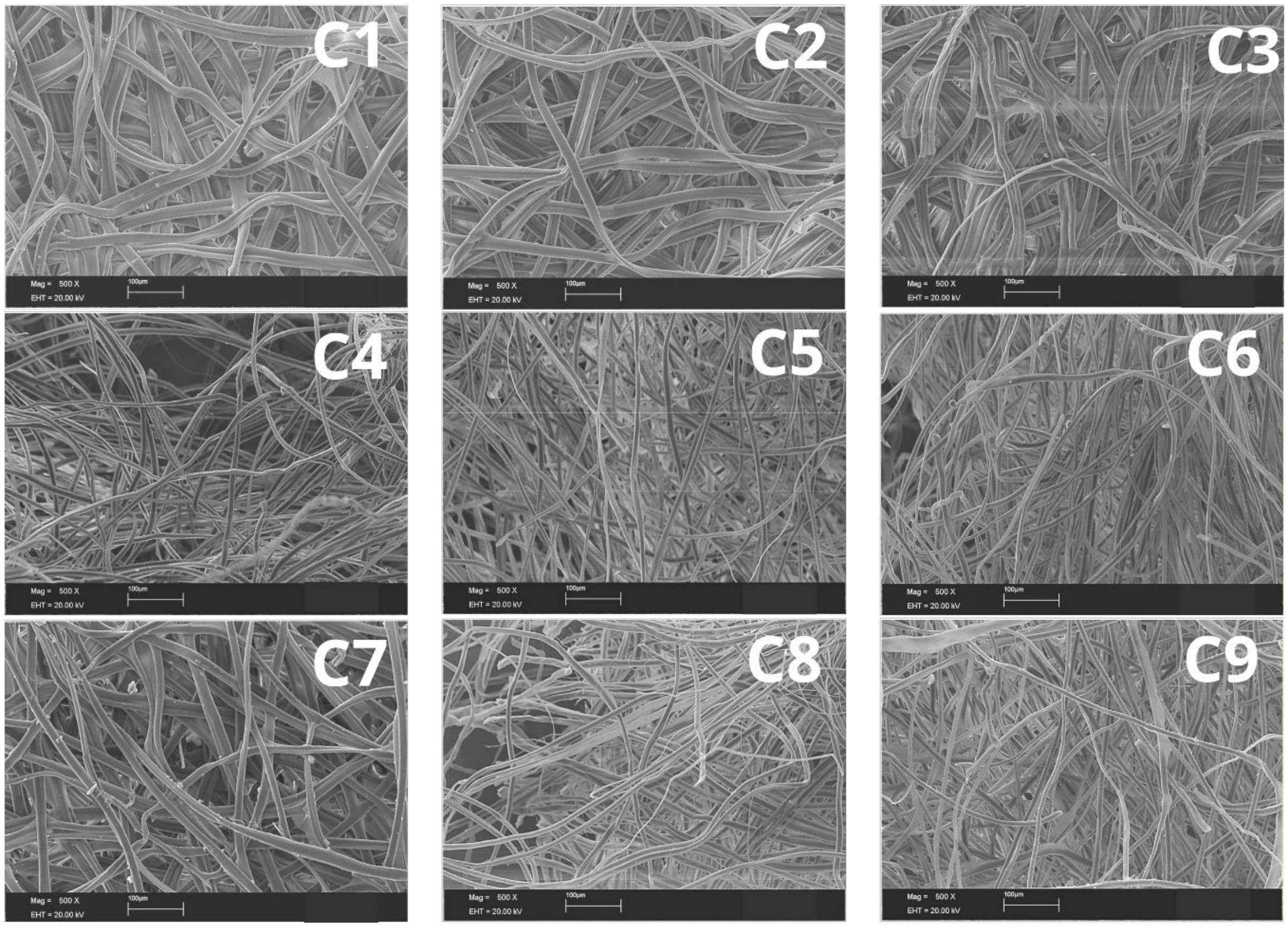

Figure S3. Micrographs of degummed process.

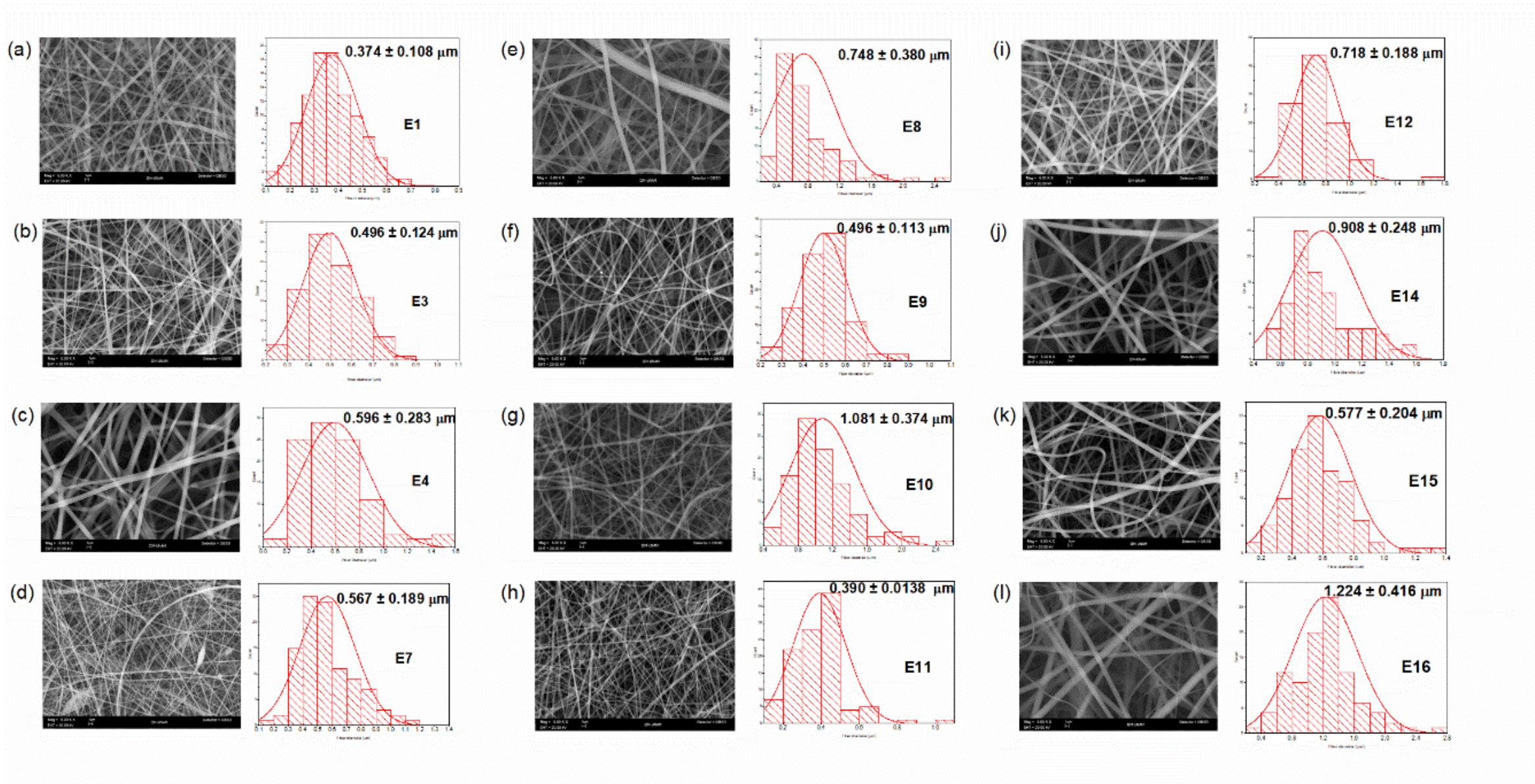

Figure S4. Distribution of the average fiber diameter of electrospun membranes. Micrographs at 5000X magnification.

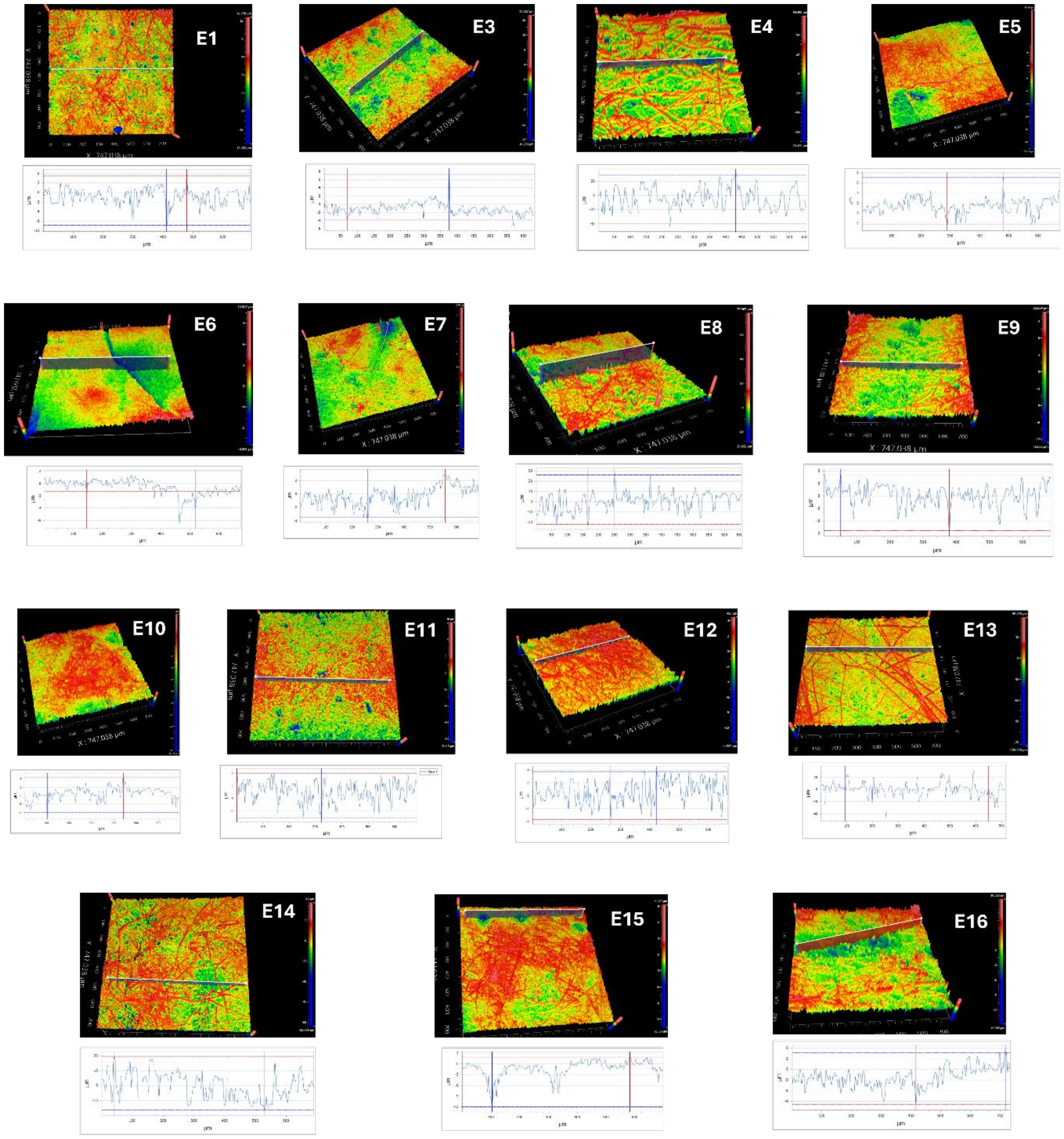

Figure S5. Surface roughness of electrospun membranes.

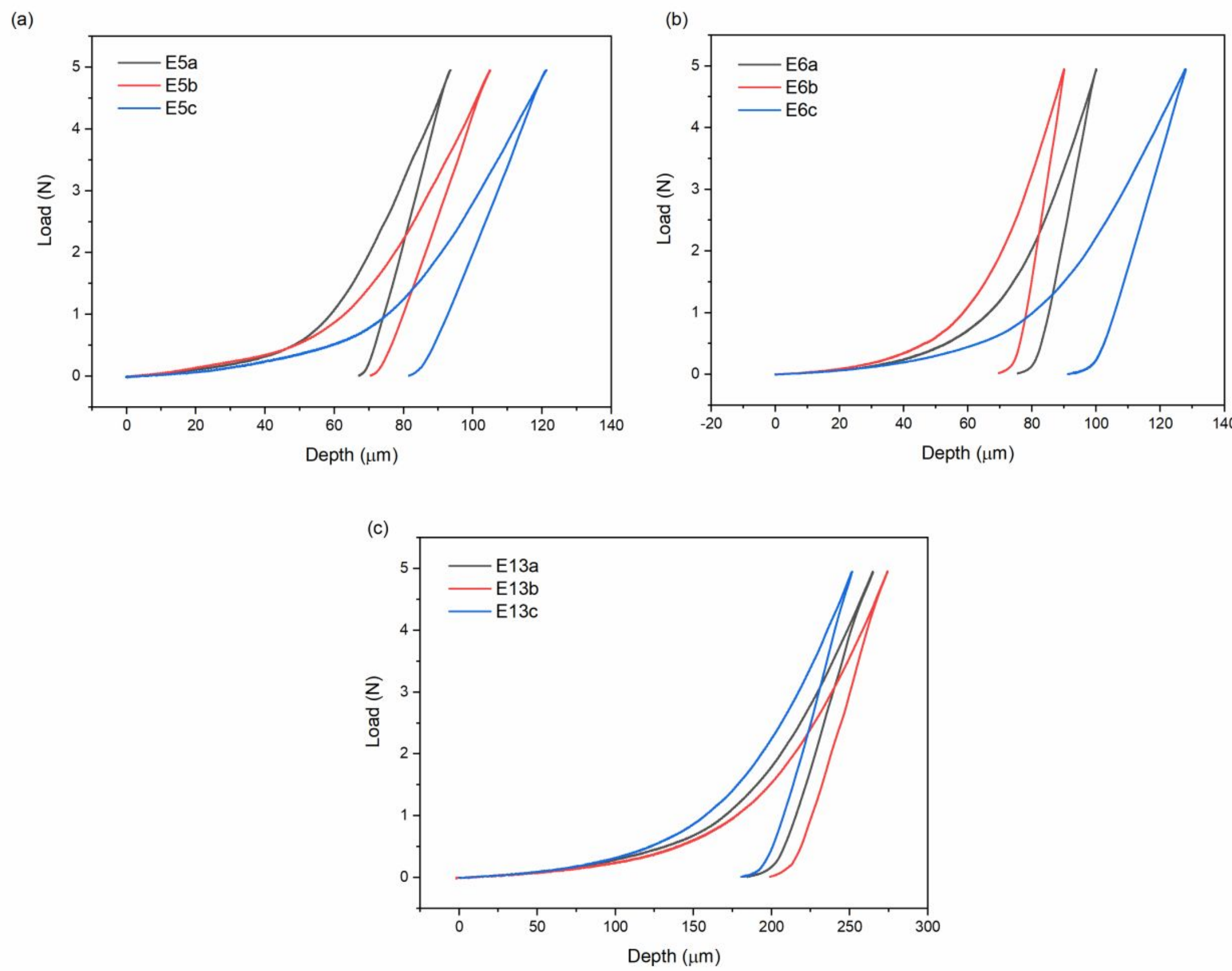

Figure S6. Microindentation curves for triplicate samples (a) E5, (b) E6, and (c) E13 electrospun membranes.

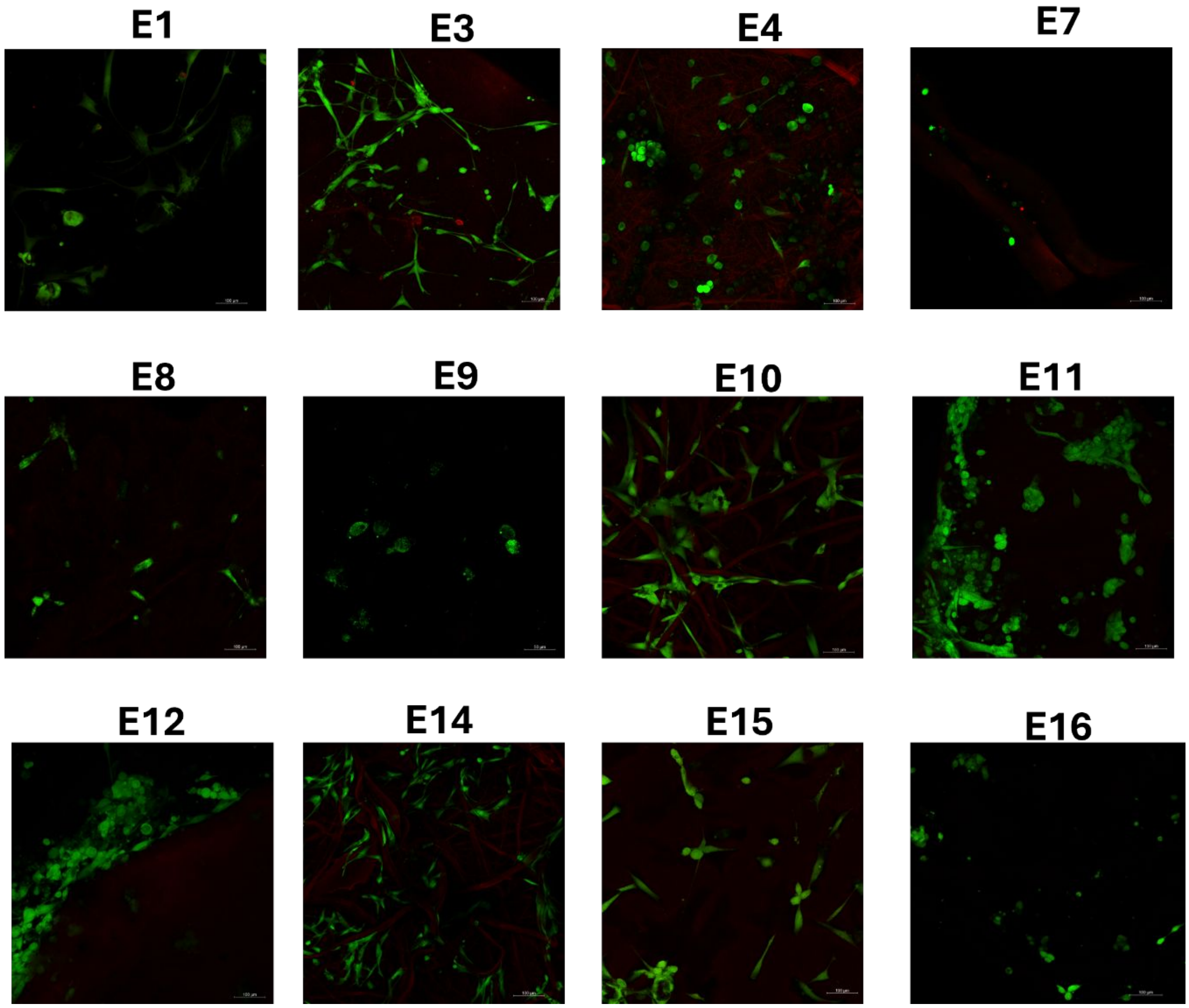

Figure S7. Micrographs of calcein assay for membranes without preserving elongated morphology. Bar scale at 100 µm
